# The Effects of Exenatide Once Weekly (EXQW) and Exenatide Twice a Day (EXBID) on Beta-Cell Function in Type 2 Diabetes: A Systematic Review and Network Meta-Analysis

**DOI:** 10.1155/2019/8083417

**Published:** 2019-10-28

**Authors:** Jie Wang, Xinye Jin, Ping An, Songyan Yu, Yiming Mu

**Affiliations:** ^1^Medicine School of Nankai University, China; ^2^Department of Endocrinology, Chinese PLA General Hospital, Beijing, China; ^3^Department of Endocrinology, Hainan Branch of PLA General Hospital, China

## Abstract

**Background:**

In patients with type 2 diabetes mellitus (T2DM) and poor glycemic control receiving metformin (MET), glucagon-like peptide-1 receptor agonists (GLP-1 RAs) are recommended as the adjunctive therapy. However, there are only a few studies involving the comparative effects of exenatide twice a day (EXBID) and exenatide once weekly (EXQW) on HOMA-*β*. This meta assessed the comparative effects of EXQW and EXBID on HOMA-*β* among T2DM patients.

**Materials and Methods:**

PubMed, Cochrane Library, and Embase databases were searched to collect randomized controlled trials (RCTs). Network meta-analysis was performed, and network diagrams were constructed to evaluate the effects. The primary outcome is HOMA-*β*, and the secondary outcomes are fasting blood glycose (FBG), glycated hemoglobin (HbA1c), and weight loss.

**Results:**

A total of 8 studies with 3506 subjects were included. Compared with other antidiabetic agents, EXQW has a greater improvement in HOMA-*β* than EXBID (weight mean difference (WMD) = ‐0.46, 95% confidence interval (CI) [-0.64, -0.28], *P* = 0.001). The effect of EXQW on HbA1c is superior to that of sitagliptin (SITA) (WMD = 0.51, 95% CI [0.03, 0.99], *P* = 0.037). The significant reduction of weight was detected for EXBID in comparison with EXQW (WMD = ‐0.73, 95% CI [-1.13, -0.33], *P* = 0.001), and no significant difference was found between EXQW and MET.

**Conclusions:**

EXQW shows a greater improvement in HOMA-*β* than EXBID. Moreover, the efficacy of EXQW on glycemic control is similar to other antidiabetic agents including EXBID. It is an advisable treatment for diabetic patients to improve HOMA-*β* and has an advantage of fewer number of injections compared with EXBID, to increase patients' adherence and quality of life.

## 1. Introduction

Type 2 diabetes mellitus (T2DM) is an increasingly prevalent chronic disease, characterized by insulin resistance and declining *β*-cell function [1]. According to the estimates of the International Diabetes Federation (IDF), the number of people with diabetes mellitus (DM) is 425 million in 2017 and will achieve 629 million in 2045 [2]. It has been one of the most challenging threats to global public health. Based on the 2018 consensus report from the American Diabetes Association (ADA) and the European Association for the Study of Diabetes (EASD), metformin (MET) and comprehensive lifestyle including weight management and physical activity are recommended as the first-line therapy [3]. In patients with T2DM and poor glycemic control receiving MET, glucagon-like peptide-1 receptor agonists (GLP-1 RAs) are recommended as the adjunctive therapy [3].

GLP-1 RAs, an established treatment option for T2DM, has been confirmed to stimulate insulin secretion and suppress glucagon secretion in a glucose-dependent manner with a low risk of hypoglycemia, indicating its efficacy and satiety [4]. Exenatide twice a day (EXBID) is the first approved GLP-1 RA with the advantages of lowering fasting, postprandial glucose concentrations, improving glycemic control, and weight loss [5, 6]. GLP-1 RAs are recommended as the first injectable treatment of T2DM considering its particular interests. In recent years, an extended-release formulation of exenatide, exenatide once weekly (EXQW), has been developed. Compared with EXBID, it has shown to sustained glycemic control and similar weight loss without increasing the risk of hypoglycemia [7–9].

It is known that patients eventually experience insufficient glycemic control with gradual *β*-cell function loss since the increased duration of T2DM. It is significant for patients with T2DM to improve *β*-cell function for better glycemic control and less glycemic fluctuation in the future. Currently, some studies have estimated the efficacy of glycemic control of EXQW or EXBID and their safety compared with other antidiabetic agents [10, 11]. EXQW, as the once weekly formulation, is expected to be related with increased adherence and compliance due to its convenience. Therefore, it is more likely to produce a better efficacy than EXBID, including improvements in *β*-cell function. However, with respect to the comparative effects of EXBID and EXQW on HOMA-*β*, a simple indicator of *β*-cell function in clinical practice, there is only one study providing head-to-head evidence within clinical trials.

Considering only one study involved in the comparative effects of EXQW and EXBID on HOMA-*β* among T2DM patients, it can be investigated by network meta-analysis using both direct and indirect evidence [12, 13]. Hence, the aim of this network meta-analysis study is to assess the comparative effects of EXQW and EXBID on HOMA-*β* among T2DM patients.

## 2. Materials and Methods

### 2.1. Search Strategy

We searched the PubMed, Cochrane Library, and Embase databases for relevant studies published in English from their inception to November 14, 2018. A combination of key words and free words were searched. The search terms used were “glucagon-like peptide-1 receptor agonists”, “exenatide”, “liraglutide”, “dulaglutide”, “semaglutide”, “albiglutide”, “placebo”, “bydureon”, “exenatide once weekly”, “*β*-cell function”, “HOMA-*β* index”, randomized controlled trial (RCT), and so forth. Reference of included trials, conference abstracts, and the previous system reviews or meta-analysis were all searched.

### 2.2. Selection of Studies

Identified studies were selected firstly on the basis of titles and abstracts by two independent authors (J.W. and XY.J.). Full articles were retrieved if a decision could not be made based on titles and abstracts. If there was disagreement between the two reviewers, a third reviewer (P.A.) were introduced. The three reviewers would hold discussions until disagreement was resolved by consensus.

### 2.3. Inclusion and Exclusion Criteria

The following literature inclusion criteria were based on the population, intervention, comparators, outcomes, and study design (PICOS) approach recommended by the Cochrane Collaboration [14]: (1) study type was RCT; (2) the study was published in English; (3) the study compared any pair involved in EXQW or EXBID; (4) study subjects were diagnosed as T2DM ranging in age more than 18 years; (5) the study included the following outcomes: HOMA-*β* index, fasting blood glycose (FBG), glycated hemoglobin (HbA1c), and weight; and (6) the full literatures could be retrieved and have sufficient data for the next extraction, including number of patients, means, and standard deviations (SD) of continuous outcomes and number of patients in each group for dichotomous outcomes. The exclusion criteria were as follows: (1) study subjects were diagnosed as type 1 diabetes mellitus (T1DM); (2) study subjects had a history of ketoacidosis, unstable, or rapidly deteriorating diabetic retinopathy, diabetic nephropathy, and diabetic neuropathy; (3) study subjects with impaired liver and kidney function or anemia; (4) studies with incomplete data; (5) non-RCTs; and (6) studies published repeatedly.

### 2.4. Data Extraction

Two researchers (J.W. and XY.J.) independently extracted the data from the included literatures according to a standardized extraction form. If there were disputes in the data extraction process, a discussion would be held with a third researcher (P.A.) until the disputes were resolved by consensus. Missing information was obtained by contacting the corresponding authors of the studies, and if not, they would be excluded.

### 2.5. Risk of Bias Assessment

Two researchers (J.W. and XY.J.) independently assessed the risk of bias of studies using Review Manager 5 [14]. The risk of bias assessment was performed according to the following six domains: sequence generation, allocation concealment, blinding, incomplete data, selective outcome reporting, and other sources of bias. Disputes were discussed with a third party (P.A.) until they were resolved by consensus.

### 2.6. Outcomes

The primary outcome was HOMA-*β* index. The secondary outcomes were FBG, HbA1c, and weight loss.

### 2.7. Statistical Analysis

A network meta-analysis was performed to compare different treatment arms using STATA software, version 15.0 [15]. A network meta-analysis synthesizes the direct and indirect evidence to compare multiple interventions and produce a ranking of treatments [16, 17]. The weight mean difference (WMD) was calculated as the effect size for continuous outcomes, and the odds ratio (OR) was calculated for dichotomous outcomes, both with a 95% confidence interval (CI). Heterogeneity of the mean difference was assessed using *Q* and *I*^2^ statistics. The node-splitting method was used to assess the consistency between direct and indirect evidence based on comparing the fit of the consistency model with the fit of an inconsistency model. There was no inconsistency if *P* value was more than 0.05. Then, the consistency model was used to analyze the data. A network meta-analysis was performed based on data augmentation approach, and the results showed as a visual inspection of forest plots [18]. Network forests were also constructed. Importantly, the figures presented the results on the basis of different design.

A network diagram was constructed by using STATA software, version 15.0 [15]. In the evidence structure plots, the nodes represented different interventions and the node size and the thickness of the lines indicated the sample size between comparisons.

A comparison-adjusted funnel plot was constructed to assess small-study effects or publication bias among the included studies. If the graph is symmetrical, this suggests no publication bias or small-study effects.

Cumulative ranking probability plots were used to show the ranking probabilities of different treatment options to guide the choice of therapy according to different treatment priorities.

Contribution plots for the included studies were performed and presented in supplemental materials.

All analyses were performed using STATA software, version 15.0 [15]. Two-sided *P* < 0.05 was considered statistically significant.

## 3. Results

**Figure 1:**
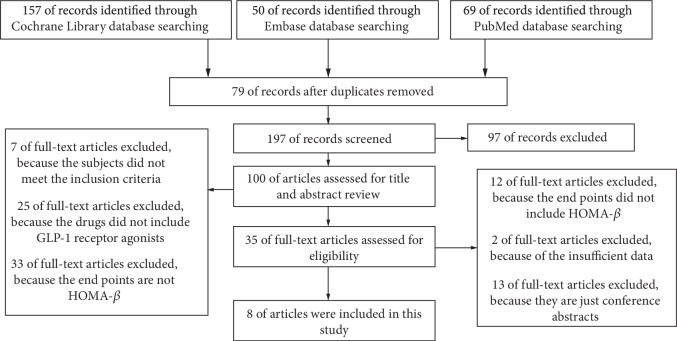
Selection of the articles included in this meta-analysis.

Among 276 identified studies, 8 studies were eligible for inclusion. Initially, a total of 276 studies were retrieved: 157 from Cochrane database, 50 from Embase database, and 69 from PubMed database. After reviewing the titles and abstracts, 35 studies were assessed for eligibility. Finally, 8 studies were included in this network meta-analysis just as described in Figure 1 [19–26]. A total of 3506 participants were included in this network meta-analysis. One study had four treatments as follows: EXQW, MET, pioglitazone (PIO), and sitagliptin (SITA) [21]. Another one had three treatments as follows: EXBID, insulin, and PIO [24]. There are 9 treatments in comparison totally. Mean age of participants ranged from 46.7 to 58.0 years. Treatment duration ranged from 16 to 56 weeks. The baseline characteristics of included studies were presented in Table 1.

The quality assessments of included studies were performed. The risk of bias was assessed by 2 reviewers (J.W. and XY.J.).The disagreement between 2 reviewers was resolved by a third researcher (P.A.) with the consensus. The overall quality of included studies in this network was good. However, as the aim of some included studies was to monitor therapeutic effects of drugs with injections, some studies were open-label. The contributions of included studies were assessed in this network meta-analysis and presented in [Supplementary-material supplementary-material-1].

### 3.1. The Evidence Network Graphs

**Figure 2:**
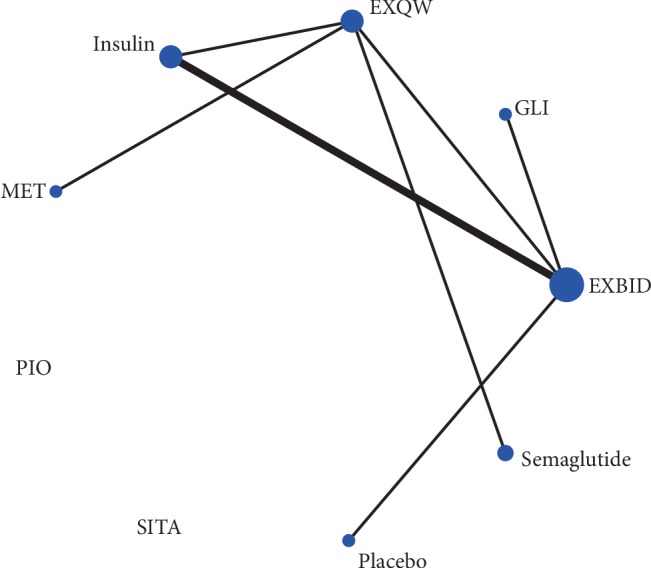
Evidence structure of eligible comparisons for network meta-analysis on HOMA-*β*.

A total of nine treatments were included in this study, and the reference was EXQW. In this current study, EXBID and EXQW are prevalent in the treatment of T2DM as shown in Figure 2. Five studies involved in EXBID and four studies involved in EXQW, whereas just one study are involved in glibenclamide (GLI), SITA, and semaglutide QW; two studies, in PIO; and three studies, in insulin.

### 3.2. Network Meta-Analysis of Consistency Model

The node-splitting method was used to test inconsistency between direct and indirect evidence. The result showed that *P* value was more than 0.05, indicating that direct evidence is in consistent with indirect evidence. Therefore, consistency model was used in this current study.

### 3.3. HOMA-*β*

**Figure 3:**
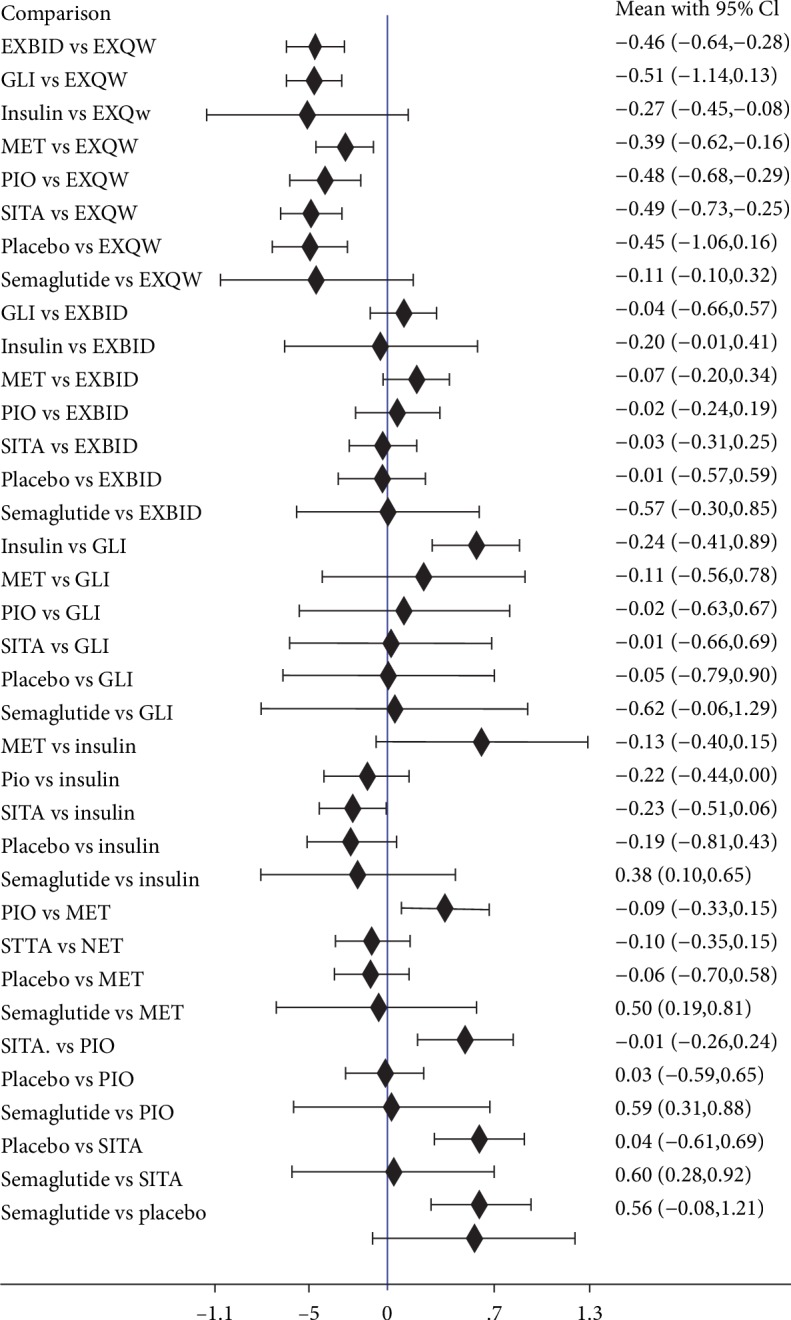
Results of network meta-analysis on HOMA-*β*.

All included studies reported the outcome of HOMA-*β*. Therefore, those were included in this network meta-analysis. Treatment comparisons were presented in [Supplementary-material supplementary-material-1] and Figure 3. Compared with EXQW, EXBID showed a smaller improvement in HOMA-*β* index (WMD = ‐0.46, 95% CI [-0.64, -0.28]).

### 3.4. FBG

All studies were included in the FBG analysis. Compared with EXQW groups, insulin had a significant reduction on FBG. However, no significant differences were found between EXBID and EXQW as shown in [Supplementary-material supplementary-material-1] and [Supplementary-material supplementary-material-1].

### 3.5. HbA1c

All studies were included in the HbA1c analysis. No significant differences were investigated between EXBID and EXQW. The results were presented in [Supplementary-material supplementary-material-1] and [Supplementary-material supplementary-material-1].

### 3.6. Weight Loss

The outcome of weight loss was reported in all included studies. In terms of weight loss, EXBID and MET both showed a greater reduction in comparison with EXQW and the results were shown in [Supplementary-material supplementary-material-1] and [Supplementary-material supplementary-material-1].

### 3.7. Funnel Plots

**Figure 4:**
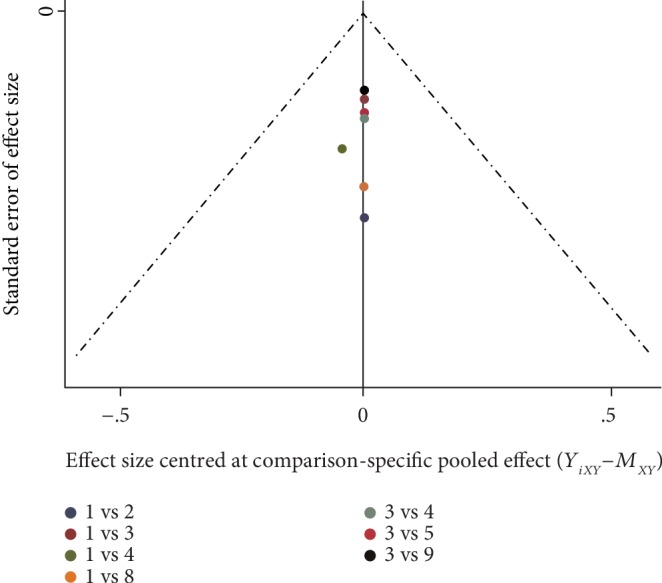
Comparison-adjusted funnel plots of HOMA-*β*. 1: EXBID; 2: GLI; 3: EXQW; 4: insulin; 5: MET; 6: PIO; 7: SITA; 8: placebo; 9: semaglutide.

There was no apparent asymmetry for the studies examining antidiabetic therapies versus EXQW from 16 to 56 weeks for any of the outcomes. The graph was symmetrical, indicating no publication bias or small research effects in the current study (Figure 4 and [Supplementary-material supplementary-material-1]).

### 3.8. Cumulative Ranking Probability Plots

**Figure 5:**
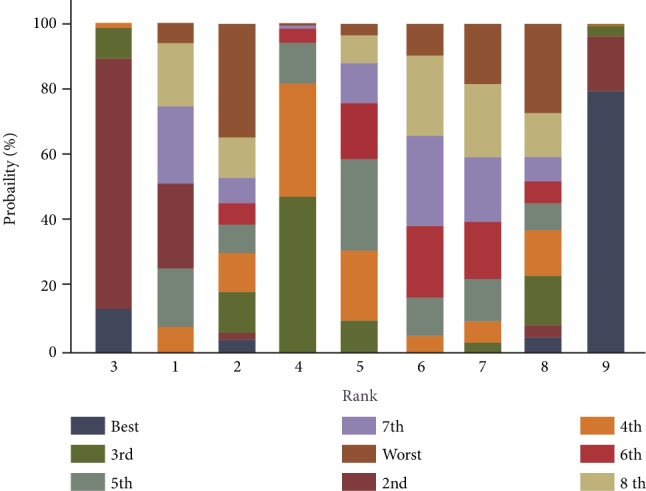
Plots of cumulative ranking probability of HOMA-*β*. 1: EXBID; 2: GLI; 3: EXQW; 4: insulin; 5: MET; 6: PIO; 7: SITA; 8: placebo; 9: semaglutide.

The ranking plots in Figure 5 and [Supplementary-material supplementary-material-1] showed the cumulative probabilities of different treatments. In comparison with other treatments, EXQW showed a significant improvement in HOMA-*β* among patients with T2DM. Compared with EXBID, EXQW showed a greater improvement in HOMA-*β*, FBG, and HbA1c.

## 4. Discussion

It is well known that patients eventually experience insufficient glycemic control with gradual *β*-cell function loss. Therefore, in addition to a focus on glycemic control, the protective effect of *β*-cell function has been paid more attention to. This current network meta-analysis analyzed the clinical relevant outcomes, including HOMA-*β*, FBG, HbA1c, and weight loss, and suggested that EXQW has a superior effectiveness compared to EXBID. The results of network meta-analysis showed that EXQW has a significant greater improvement in HOMA-*β* with the fewer subcutaneous injections in comparison with EXBID.

Given only one study involved in head-to-head clinical trials about EXQW and EXBID, network meta-analysis was conducted to analyze the indirect comparisons. In comparison with traditional meta-analysis, network meta-analysis can assess estimates of treatment efficacy of multiple treatment options by using direct and indirect evidence. In addition, it can synthesize data effects to rank the treatment options on different outcomes.

In our meta-analysis, EXQW showed a favorable effect on outcomes. The results were consistent with previous studies. Macconell et al. reported that EXQW not only produced a clinical improvement in *β*-cell function but also significantly reduced HbA1c and weight [27]. In some randomized trials, EXQW was reported to show a significantly greater reduction in HbA1c and weight than SITA, PIO, and insulin. Additionally, in a 30-week, randomized, open-label study (DURATION-1), EXQW presented superiority to EXBID in reducing HbA1c among T2DM patients. However, EXQW was not superior to EXBID in weight loss, which was in line with our findings [7, 8, 20, 28].

Importantly, it is well known that GLP-1 plays a significant role in the homeostasis of *β*-cell mass by both stimulating *β*-cell proliferation and protecting against apoptosis, since it can activate some key kinases, including PKA, PI3-kinase, and ERK1/2 [29]. These kinases are all involved in *β*-cell proliferation. Exenatide as a 39-amino acid synthetic version of exendin-4 shares 53% structural homology to human GLP-1, so its biological properties are similar to human GLP-1. It can bind the GLP-1 receptor and have the effects of GLP-1. Because glycine occurs in the penultimate N-terminal position (Ala8) instead of alanine, it can be resistant to degradation by DPP-4 and have a longer half-life than GLP-1, which are the advantages of being antihyperglycemic agent [30]. A positive effect of exenatide on insulin secretion, *β*-cell proliferation, and survival has been confirmed [31, 32]. Currently, exenatide has been developed in two formulation, including EXBID and EXQW. EXBID is a short-acting formulation with a mean terminal half-life of 2.4 h, in which exenatide is dissolved in a sterile solution (250 *μ*g/mL). Because of its half-life, EXBID is administered twice daily. Compared with EXBID, EXQW is an extended-release formulation of exenatide, which encapsulates exenatide in poly-microspheres. Hence, it can release the drug more slowly and maintain the therapeutic concentration for a longer time than EXBID.

The superiority of EXQW in HOMA-*β* over EXBID may be partly explained by the following mechanisms. Firstly, the gradual release of EXQW from the microspheres could help T2DM patients reduce the numbers of subcutaneous injections. Due to the convenience of the extended-release formulation, it may improve patient adherence and quality of life, leading to a better glycemic control compared with the short-acting GLP-1 RAs. In addition, considering that the same active ingredient is contained in EXBID and EXQW, the more favorable effect of EXQW may be explained by their different formulation, which might have an impact on the plasma concentrations in peak or steady state. Compared with the inevitable fluctuation of plasma exenatide concentrations in EXBID formulation, the concentrations in EXQW formulation are steadier during the day, probably contributing to an improvement in HOMA-*β* among T2DM patients [8, 33].

Our study has several strengths. The study methods were systematic and exhaustive. Funnel plots were constructed to identify publication bias because of small-study effects, which probably result in greater treatment effects than large studies [34, 35]. Additionally, all possible treatment comparisons were mapped by using a network meta-analysis, which can use not only direct evidence but also indirect evidence to increase the total sample sizes. Though the results are heterogeneous to some extent, a random effects model was used, which takes variations into account at the study level. Another key strength is cumulative ranking probability plots, which were constructed, in order to rank the treatment options statistically. Meanwhile, to our best knowledge, this is the first network meta-analysis study to analyze the improvement effects of EXQW in HOMA-*β* index compared with its short formulation EXBID and other antidiabetic agents, including MET, GLI, insulin, PIO, SITA, semaglutide, and placebo.

Several limitations, however, should be noted. First, only RCTs in English were included, potentially inducing publication bias or selection bias. Though all included studies were RCTs, several studies were unclear in blinding of outcome assessments and, hence, detection bias or confounding might be present. Secondly, due to the different durations of treatment, the existence of heterogeneity is inevitable. Finally, none of the trials included was especially designed to evaluate the effects of the above-mentioned drugs on HOMA-*β* index. Thus, the results should be draw with caution.

## 5. Conclusions

EXQW as an extended-release formulation has an improvement in HOMA-*β*. And its favorable effect on HOMA-*β* is superior to insulin and EXBID. Importantly, the effect of EXQW on glycemic control is comparable to other antidiabetic agents. Taking the above results into account, EXQW can be suggested as a kind of adjunctive treatment of T2DM to achieve well glycemic control and protect *β*-cell function. However, studies related with EXQW are limited. More clinical trials or further evidence are necessary to identify the favorable effects of EXQW and provide more evidence to guide the choice of treatment for T2DM according to patient's different condition.

## Figures and Tables

**Table 1:** Baseline characteristics of the included studies.

First author, study acronym	Year published	Number of participants	Intervention	Prestudy OAD treatment *N* (%)			Duration of treatments (weeks)	Baseline characteristics				
				None	Monotherapy	Combination therapy		Age (years) mean (SD)	Duration of T2DM (years)	HbA1c (%)	FBG (mmol/L)	BMI
1 (Derosa)	2009	60	EXBID		60 (100)		26	57 ± 8		8.8 ± 0.7	8 ± 0.05	28.7 ± 1.5
1 (Derosa)		61	Glibenclamide		61 (100)		26	56 ± 7		8.9 ± 0.8	8.2 ± 0.06	28.5 ± 1.4
2 (Diamant)	2010	233	EXQW		164 (70)	69 (30)	26	58 ± 10	8 ± 6	8.3 ± 1.1	9.9 ± 2.5	32 ± 5
2 (Diamant)		223	Insulin glargine		157 (70)	66 (30)	26	58 ± 9	7.8 ± 6	8.3 ± 1.0	9.7 ± 2.7	32 ± 5
3 (Russell)	2012	248	EXQW	248 (100)			26	54 ± 11	2.7 ± 3.2	8.5 ± 1.2	9.9 ± 2.9	31.4 ± 5.3
3 (Russell)		246	MET	246 (100)			26	54 ± 11	2.6 ± 3.6	8.6 ± 1.2	10.0 ± 3.4	30.7 ± 5.5
		163	PIO	163 (100)			26	55 ± 11	2.7 ± 3.7	8.5 ± 1.2	9.8 ± 3.0	31.1 ± 5.3
		163	SITA	163 (100)			26	52 ± 11	2.7 ± 3.7	8.5 ± 1.3	9.7 ± 2.6	31.8 ± 5.4
4 (Derosa)	2013	84	EXBID		84 (100)		26	57.3 ± 7.7	7.6 ± 2.8	8.1 ± 0.8	7.8 ± 1.0	31.9 ± 1.7
4 (Derosa)		83	Placebo		83 (100)		26	56.7 ± 7.3	7.8 ± 3.1	7.9 ± 0.6	7.7 ± 0.8	31.7 ± 1.5
5 (Linong)	2013	338	EXBID				26	56 ± 10	8.6 ± 6	8.7 ± 1.0	9.4 ± 2.7	26.7 ± 3.4
5 (Linong)		340	EXQW				26	55 ± 11	7.7 ± 5.1	8.7 ± 1.0	9.1 ± 2.4	26.4 ± 3.7
6 (W.XU)	2015	142	EXBID	142 (100)			48	50 ± 9.5		8.0 ± 1.2	8.9 ± 2.4	26.1 ± 3.6
6 (W.XU)		138	Insulin	138 (100)			48	51 ± 9.4		8.1 ± 1.2	8.9 ± 2.3	25.6 ± 3.5
		136	PIO	136 (100)			48	50 ± 9.3		8.0 ± 1.2	9.2 ± 2.3	25.9 ± 3.5
7 (YIN)	2018	19	EXBID		19 (100)		16	46.74 ± 2.31	6.37 ± 0.99	8.01 ± 0.21	8.65 ± 0.53	28.03 ± 0.50
7 (YIN)		20	Insulin		20 (100)		16	49.45 ± 2.17	4.35 ± 0.68	8.35 ± 0.24	8.83 ± 0.48	27.08 ± 0.52
8 (Ahmann)	2018	404	Semaglutide QW				56	56.4 (20-82)	9.0 (0.4-37.1)	8.4 (6.7-11.1)		34.0 (21.0-72.8)
8 (Ahmann)		405	EXQW				56	56.7 (21-83)	9.4 (0.3-54.0)	8.3 (6.5-11.2)		33.6 (21.2-55.8)

## Additional Points

*Highlights*. This is the first network meta-analysis study to analyze the improvement effects of EXQW in HOMA-*β* index compared with its short formulation EXBID and other antidiabetic agents. The study methods were systematic to provide more evidence to guide the choice of EXBID and EXQW for T2DM.

## Supplementary Materials

Supplementary Materials Table S1: results of a random effects model of HOMA-B. Table S2: results of a random effects model of FBG. Table S3: results of a random effects model of HbA1c. Table S4: results of a random effects model of weight loss. Figure S1: evidence structure of eligible comparisons for network meta-analysis of the secondary outcomes (FBG, HbA1c, and weight loss). Figure S2: results of network meta-analysis of the secondary outcomes (FBG, HbA1c, and weight loss). Figure S3: comparison-adjusted funnel plots of the secondary outcomes (FBG, HbA1c, and weight loss). Figure S4: plots of cumulative ranking probability of the secondary outcomes (FBG, HbA1c, and weight loss). Figure S5: the contribution plots of included studies. Figure S6: node-splitting plots of outcomes.
