# Supplementary material for: The Effects of Exenatide Once Weekly (EXQW) and Exenatide Twice a Day (EXBID) on Beta-Cell Function in Type 2 Diabetes: A Systematic Review and Network Meta-Analysis
Source: J Diabetes Res. 2019 Oct 28;2019:8083417. doi: 10.1155/2019/8083417 (PMC6854923; doi:10.1155/2019/8083417)
Supplement: Supplementary Materials — Table S1: results of a random effects model of HOMA-B. Table S2: results of a random effects model of FBG. Table S3: results of a random effects model of HbA1c. Table S4: results of a random effects model of weight loss. Figure S1: evidence structure of eligible comparisons for network meta-analysis of the secondary outcomes (FBG, HbA1c, and weight loss). Figure S2: results of network meta-analysis of the secondary outcomes (FBG, HbA1c, and weight loss). Figure S3: comparison-adjusted funnel plots of the secondary outcomes (FBG, HbA1c, and weight loss). Figure S4: plots of cumulative ranking probability of the secondary outcomes (FBG, HbA1c, and weight loss). Figure S5: the contribution plots of included studies. Figure S6: node-splitting plots of outcomes. [file 8083417.f1.pdf]

Supplemental material

Table S1: Results of a random-effects model of HOMA-B

Intervention	Coef.	SD	z	P>|z|	[95% CI]
EXBID	-0.46	0.91	-5.07	0.001	-0.64, -0.28
Glibenclamide 2.5mg	-0.51	0.32	-1.55	0.121	-1.14, 0.13

insulin	-0.27	0.09	-2.81	0.005	-0.45, -0.08
MET	-0.39	0.12	-3.38	0.001	-0.62, -0.16
PIO	-0.48	0.10	-4.85	0.001	-0.68, -0.29
SITA	-0.49	0.12	-4.01	0.001	-0.73, -0.25
placebo	-0.45	0.31	-1.45	0.147	-1.06, 0.16
Semaglutide QW	0.11	0.11	1.03	0.303	-0.10, 0.32

Table S2: Results of a random-effects model of FBG

Intervention	Coef.	SD	z	P>|z|	[95% CI]
EXBID	0.20	0.46	0.44	0.658	-0.70, 1.10
Glibenclamide 2.5mg	-0.07	0.75	-0.09	0.925	-1.54, 1.40
insulin	-0.02	0.49	-0.04	0.968	-0.97, 0.93
MET	0.34	0.61	0.56	0.574	-0.85, 1.54
PIO	-0.21	0.50	-0.42	0.675	-1.19, 0.77
SITA	1.23	0.63	1.96	0.050	0.002, 2.48
placebo	0.71	0.79	0.91	0.365	-0.83, 2.26
Semaglutide QW	-0.80	0.66	-1.22	0.222	-2.08, 0.48

Table S3: Results of a random-effects model of HbA1c

Intervention	Coef.	SD	z	P>|z|	[95% CI]
EXBID	0.12	0.18	0.66	0.511	-0.25, 0.48
Glibenclamide 2.5mg	-0.08	0.32	-0.24	0.808	-0.71, 0.55
insulin	0.14	0.18	0.74	0.457	-0.22, 0.49
MET	0.18	0.24	0.74	0.460	-0.29, 0.65
PIO	0.16	0.19	0.83	0.407	-0.22, 0.54
SITA	0.51	0.24	2.09	0.037	0.03, 0.99
placebo	0.52	0.31	1.66	0.097	-0.09, 1.13
Semaglutide QW	-0.60	0.25	-2.40	0.016	-1.09, -0.11

Table S4: Results of a random-effects model of weight loss

Intervention	Coef.	SD	z	P>|z|	[95% CI]
EXBID	-0.73	0.20	-3.56	0.001	-1.13, -0.33
Glibenclamide 2.5mg	4.47	1.58	2.84	0.005	1.38, 7.57
insulin	3.99	0.24	16.95	0.001	3.53, 4.45
MET	-0.07	0.28	-0.25	0.799	-0.61, 0.47
PIO	3.27	0.29	11.25	0.001	2.70, 3.84
SITA	1.13	0.35	3.19	0.001	0.44, 1.82
placebo	1.27	1.46	0.87	0.382	-1.58, 4.13

Semaglutide QW	-3.70	0.41	-9.02	0.001	-4.50, -2.90

Figure S1: Evidence structure of eligible comparisons for network meta-analysis of the secondary outcomes (FBG, HbA1c, Weight loss)

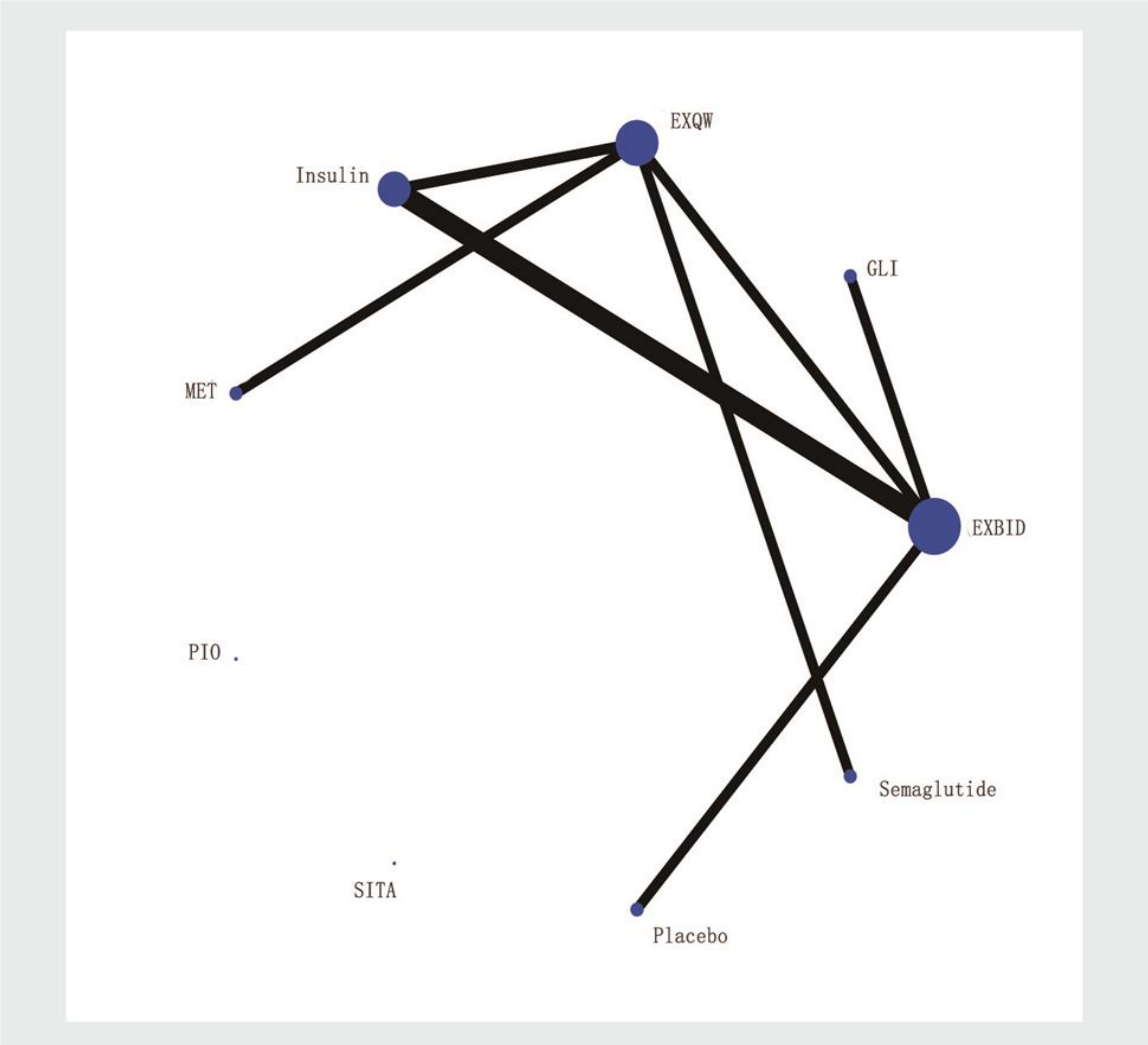

A: FBG

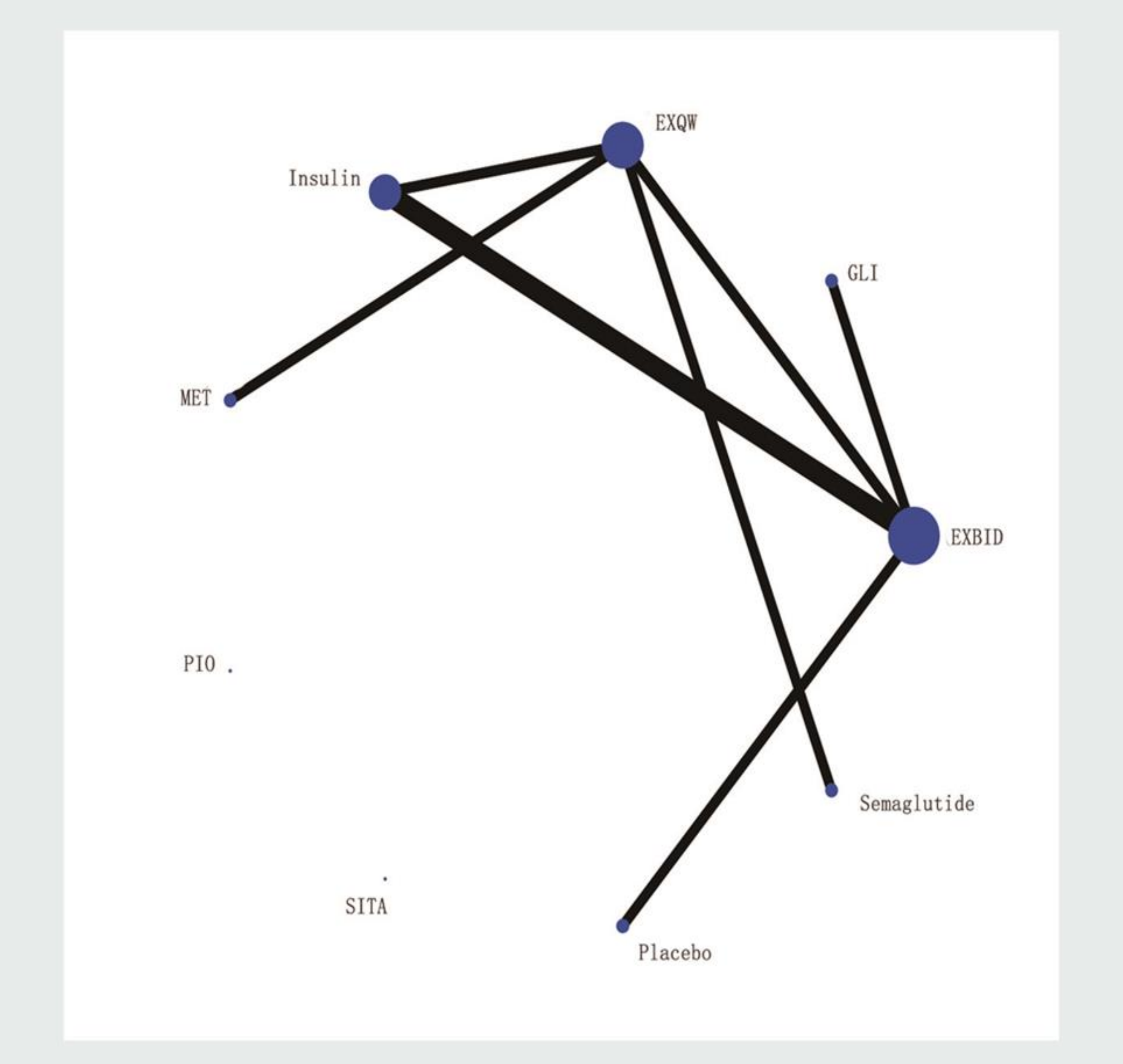

B: HbA1c

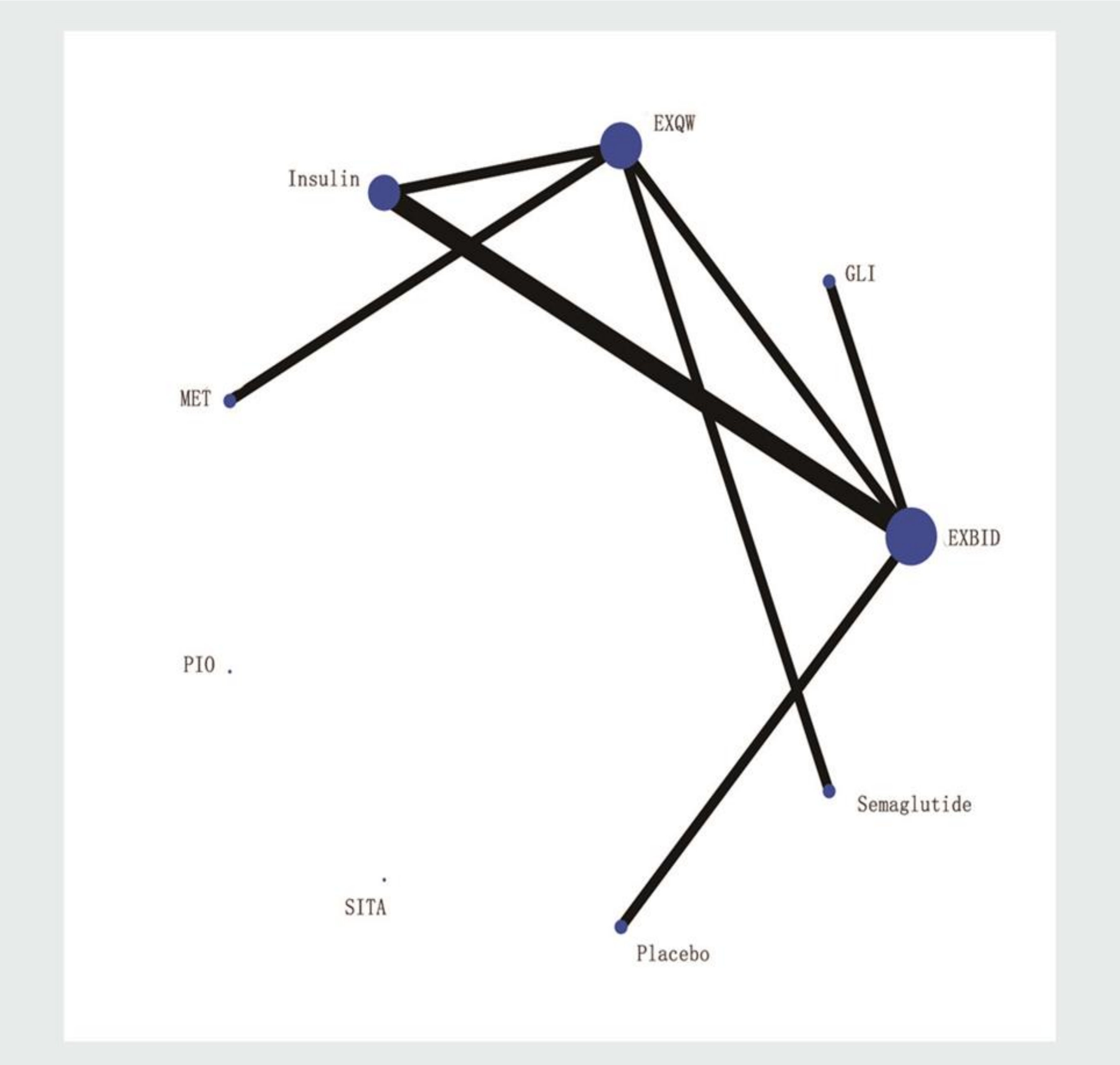

451 **C: Weight loss**

Figure S2: Results of network meta-analysis of the secondary outcomes (FBG, HbA1c, Weight loss).

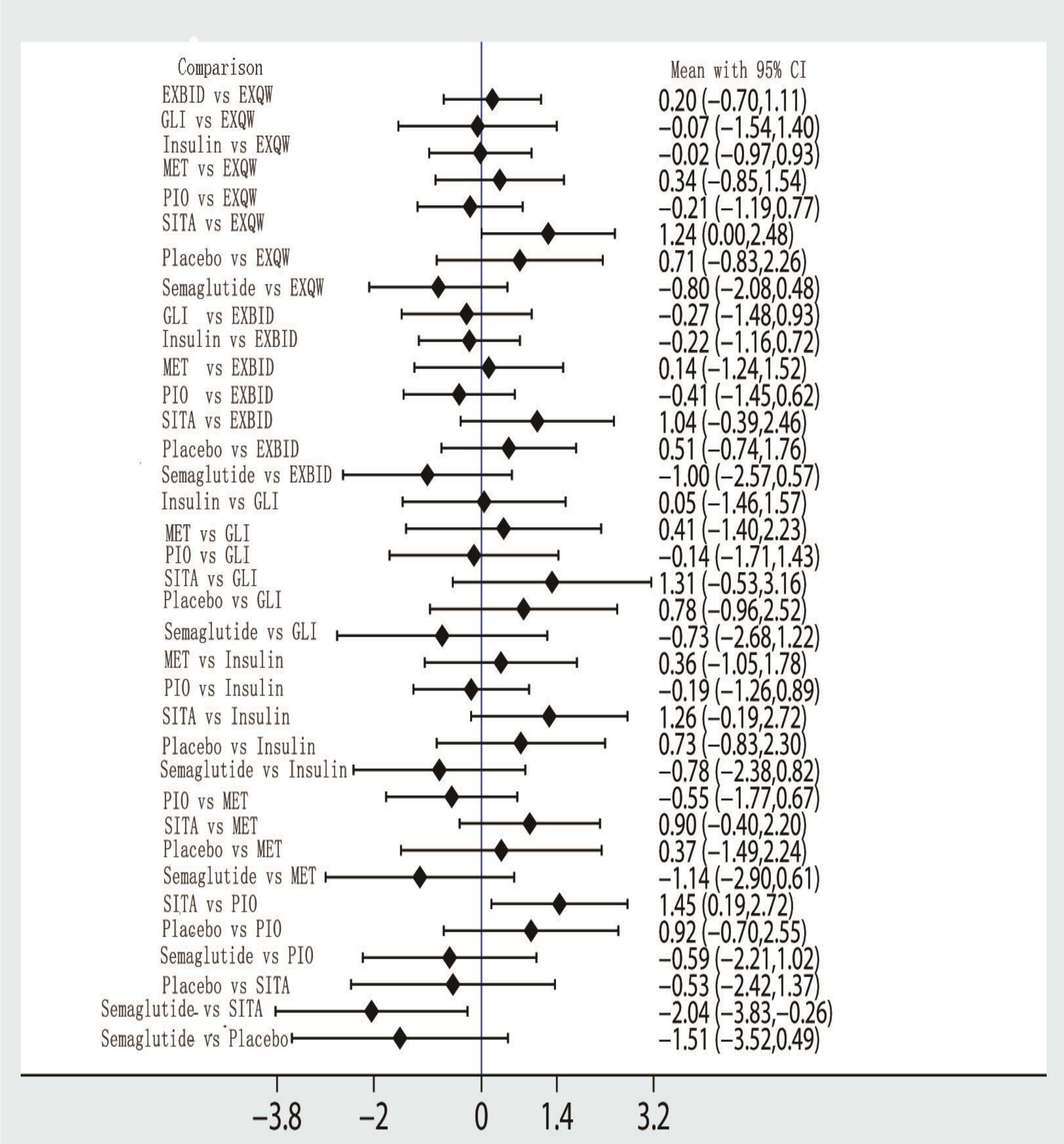

A: FBG

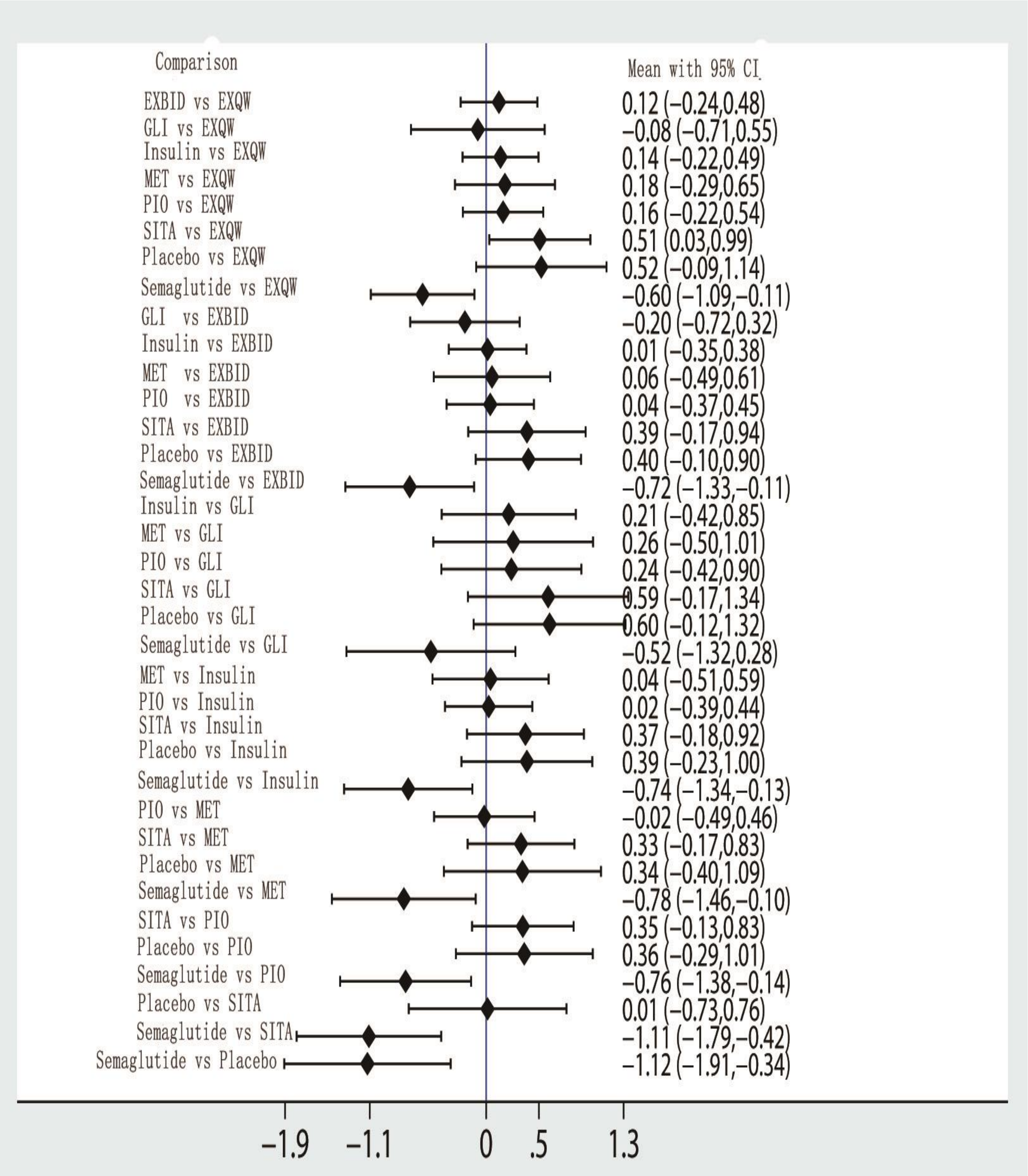
Comparison
Mean with 95% CI
EXBID vs EXQW
0.12 (−0.24,0.48)
GLI vs EXQW
−0.08 (−0.71,0.55)
Insulin vs EXQW
0.14 (−0.22,0.49)
MET vs EXQW
0.18 (−0.29,0.65)
PIO vs EXQW
0.16 (−0.22,0.54)
SITA vs EXQW
0.51 (0.03,0.99)
Placebo vs EXQW
0.52 (−0.09,1.14)
Semaglutide vs EXQW
−0.60 (−1.09,−0.11)
GLI vs EXBID
−0.20 (−0.72,0.32)
Insulin vs EXBID
0.01 (−0.35,0.38)
MET vs EXBID
0.06 (−0.49,0.61)
PIO vs EXBID
0.04 (−0.37,0.45)
SITA vs EXBID
0.39 (−0.17,0.94)
Placebo vs EXBID
0.40 (−0.10,0.90)
Semaglutide vs EXBID
−0.72 (−1.33,−0.11)
Insulin vs GLI
0.21 (−0.42,0.85)
MET vs GLI
0.26 (−0.50,1.01)
PIO vs GLI
0.24 (−0.42,0.90)
SITA vs GLI
0.59 (−0.17,1.34)
Placebo vs GLI
0.60 (−0.12,1.32)
Semaglutide vs GLI
−0.52 (−1.32,0.28)
MET vs Insulin
0.04 (−0.51,0.59)
PIO vs Insulin
0.02 (−0.39,0.44)
SITA vs Insulin
0.37 (−0.18,0.92)
Placebo vs Insulin
0.39 (−0.23,1.00)
Semaglutide vs Insulin
−0.74 (−1.34,−0.13)
PIO vs MET
−0.02 (−0.49,0.46)
SITA vs MET
0.33 (−0.17,0.83)
Placebo vs MET
0.34 (−0.40,1.09)
Semaglutide vs MET
−0.78 (−1.46,−0.10)
SITA vs PIO
0.35 (−0.13,0.83)
Placebo vs PIO
0.36 (−0.29,1.01)
Semaglutide vs PIO
−0.76 (−1.38,−0.14)
Placebo vs SITA
0.01 (−0.73,0.76)
Semaglutide vs SITA
−1.11 (−1.79,−0.42)
Semaglutide vs Placebo
−1.12 (−1.91,−0.34)
−1.9
−1.1
0
.5
1.3

B: HbA1c

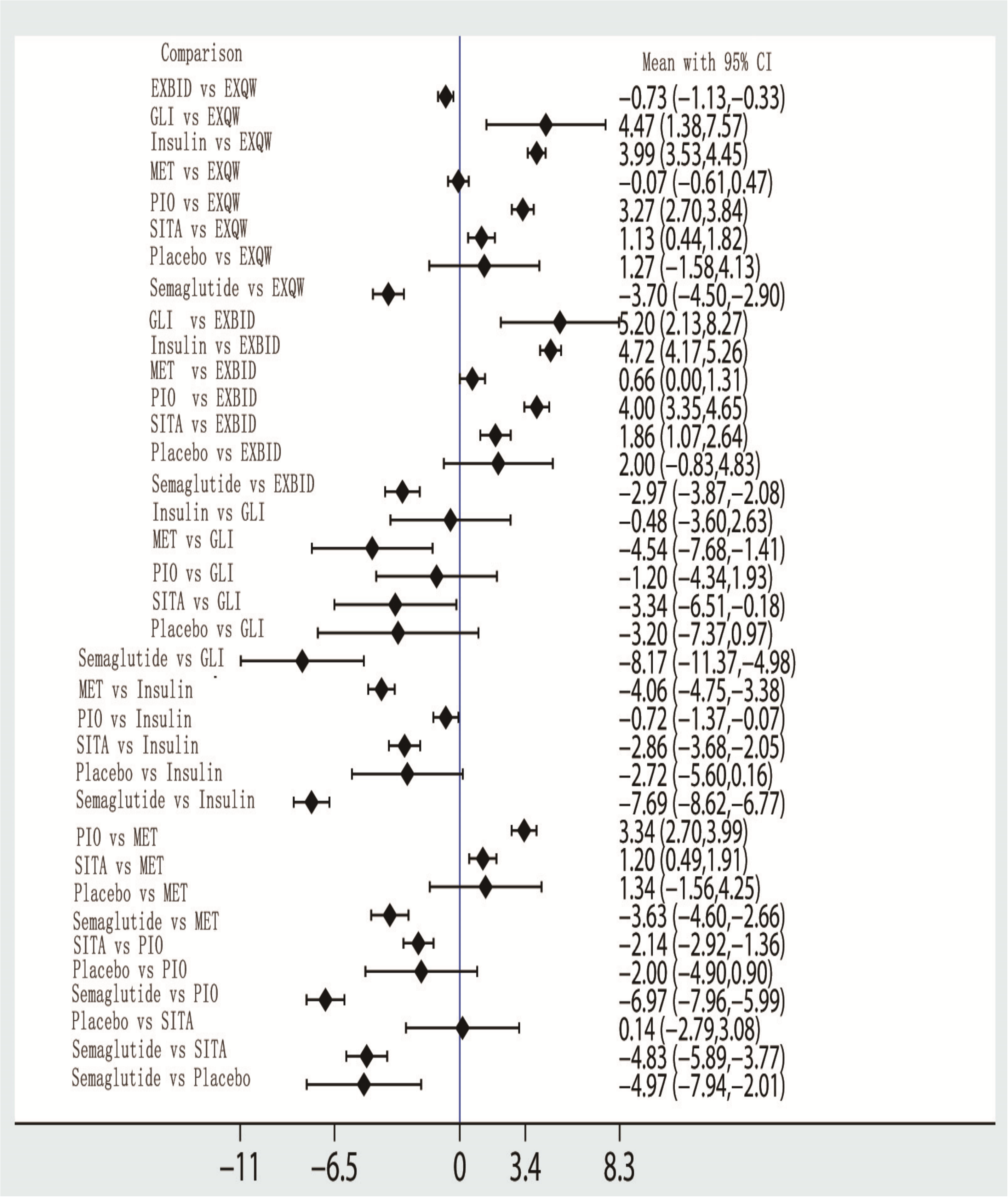

C: Weight loss .

Figure S3: Comparison-adjusted funnel plots of the secondary outcomes (FBG, HbA1c, Weight loss)

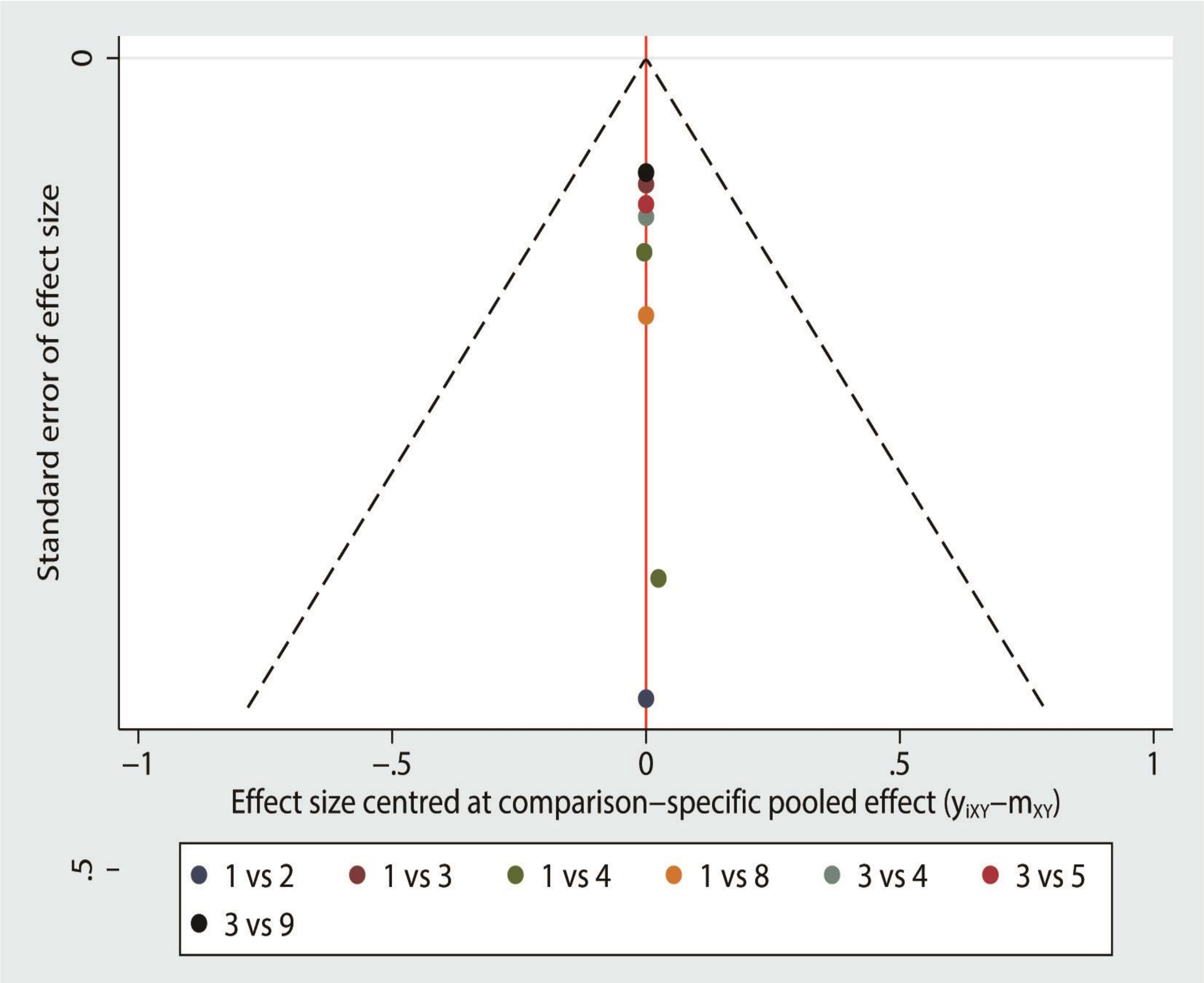

A: FBG

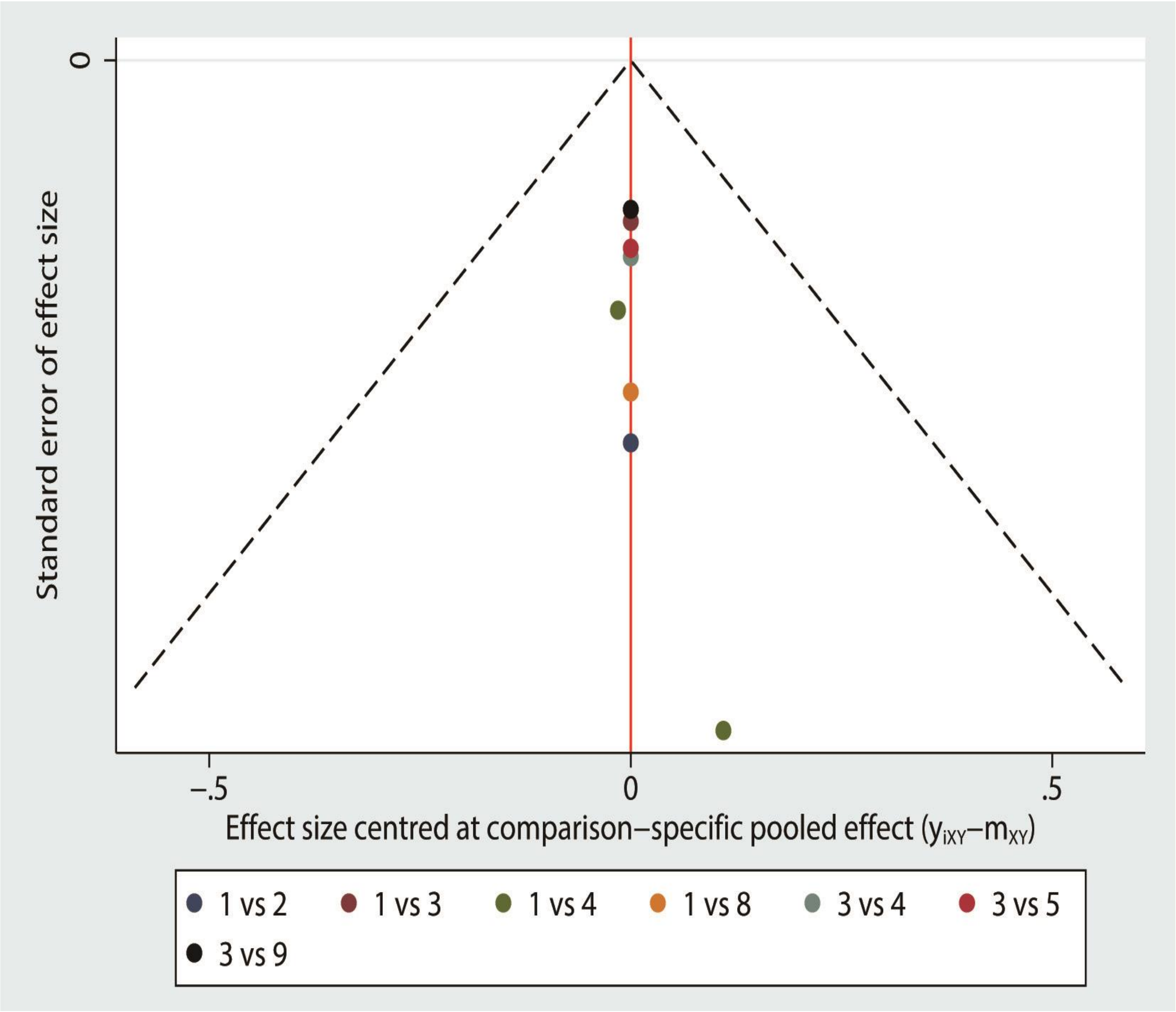
0
Standard error of effect size
−.5
0
.5
Effect size centred at comparison−specific pooled effect (yiXY−mXY)
1 vs 2
1 vs 3
1 vs 4
1 vs 8
3 vs 4
3 vs 5
3 vs 9

B: HbA1c

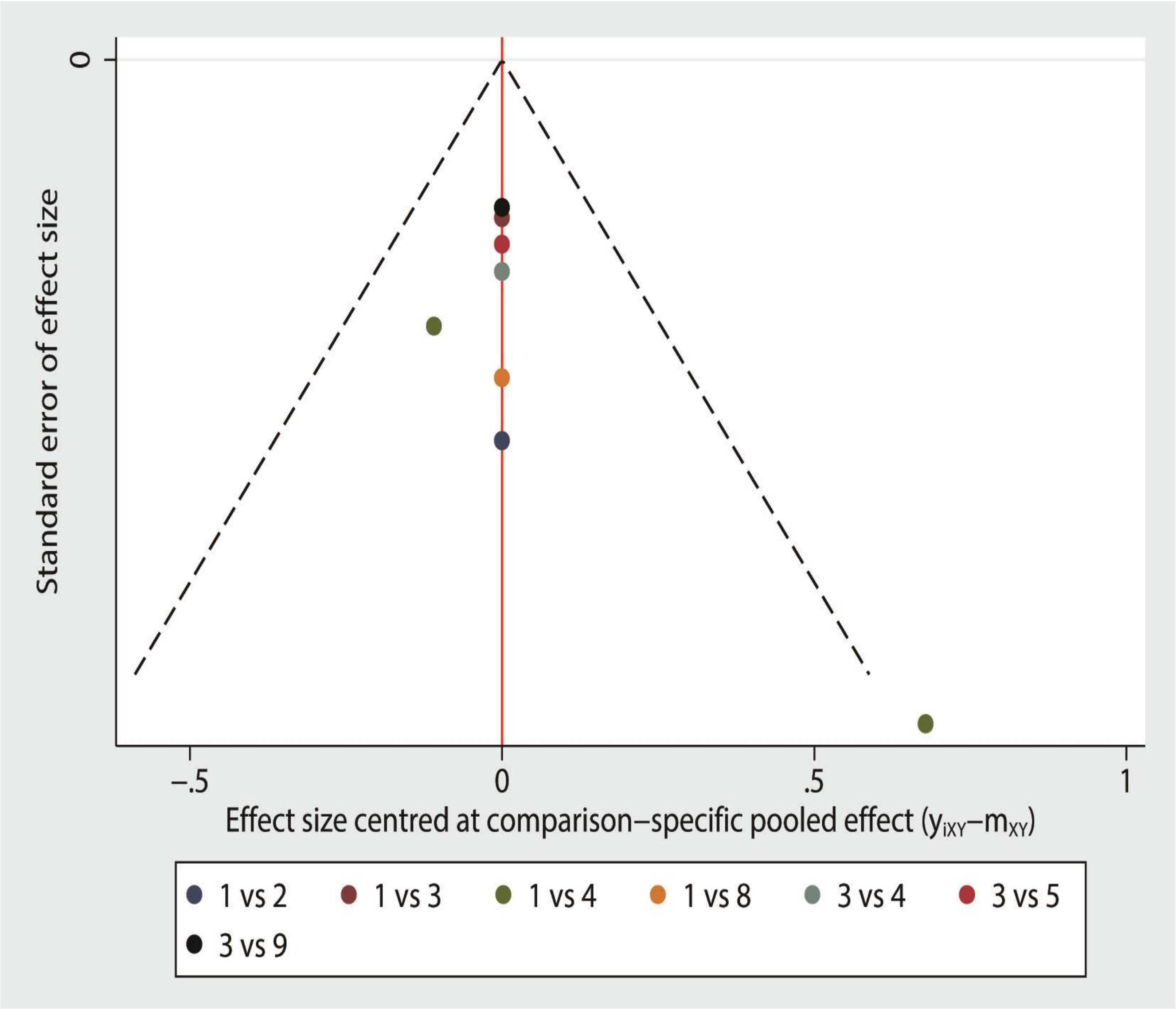

C: Weight loss

Figure S4: Plots of cumulative ranking probability of the secondary outcomes (FBG, HbA1c, Weight loss)

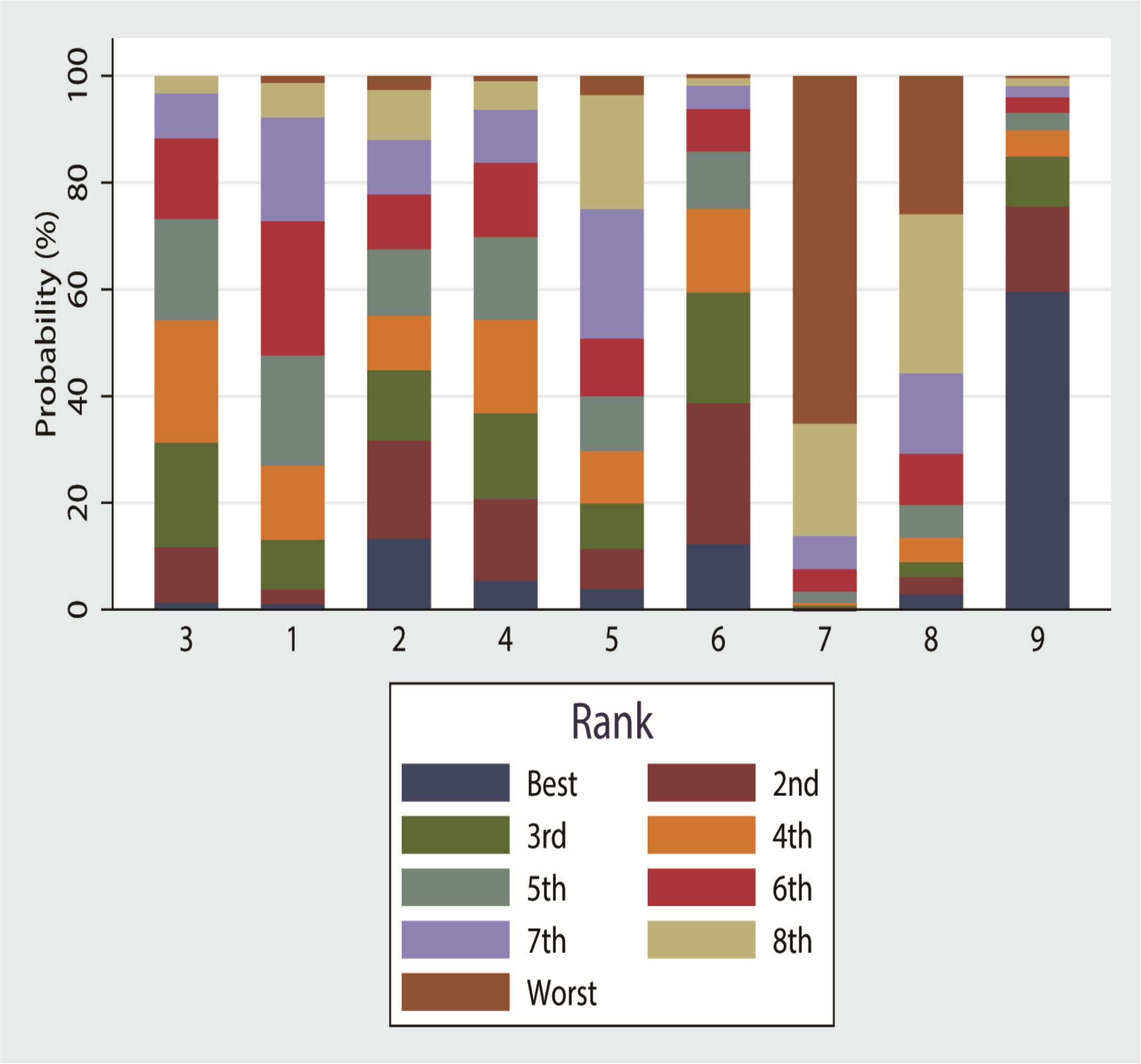

A: FBG

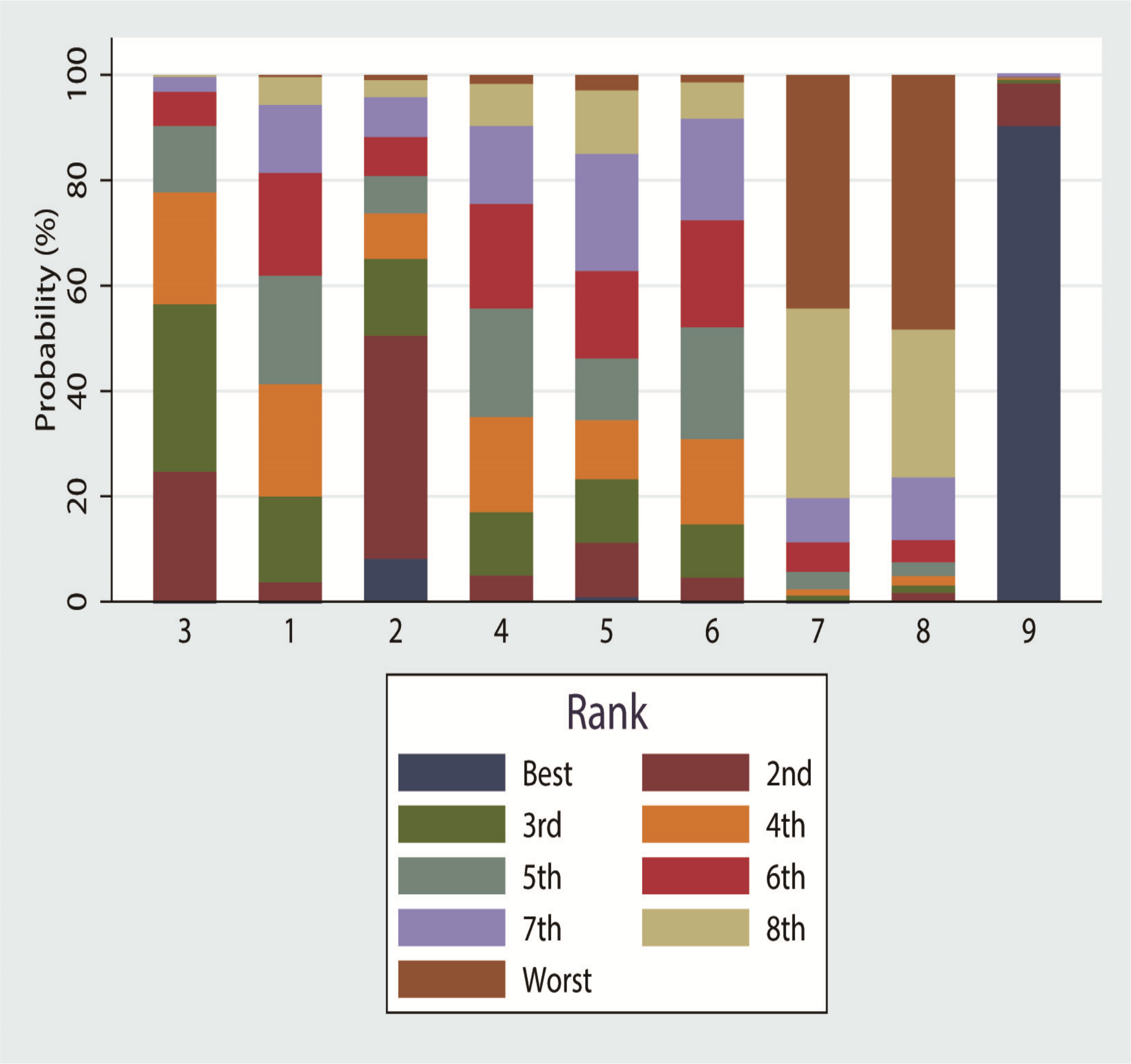
Probability (%)
0
20
40
60
80
100
3
1
2
4
5
6
7
8
9
Rank
Best
2nd
3rd
4th
5th
6th
7th
8th
Worst

B: HbA1c

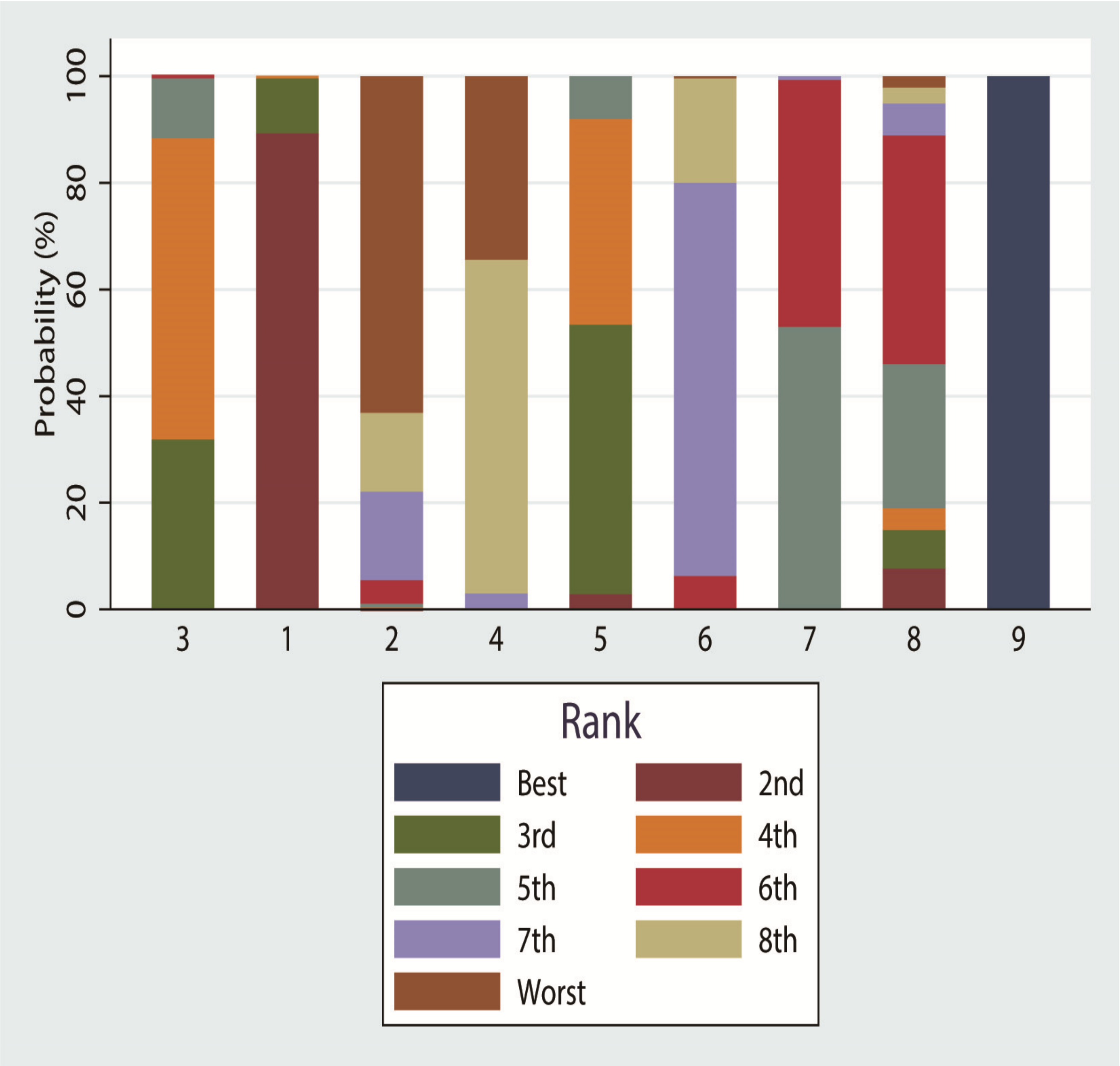

498 **C: Weight loss**

499

Figure S5: The contribution plots of included studies

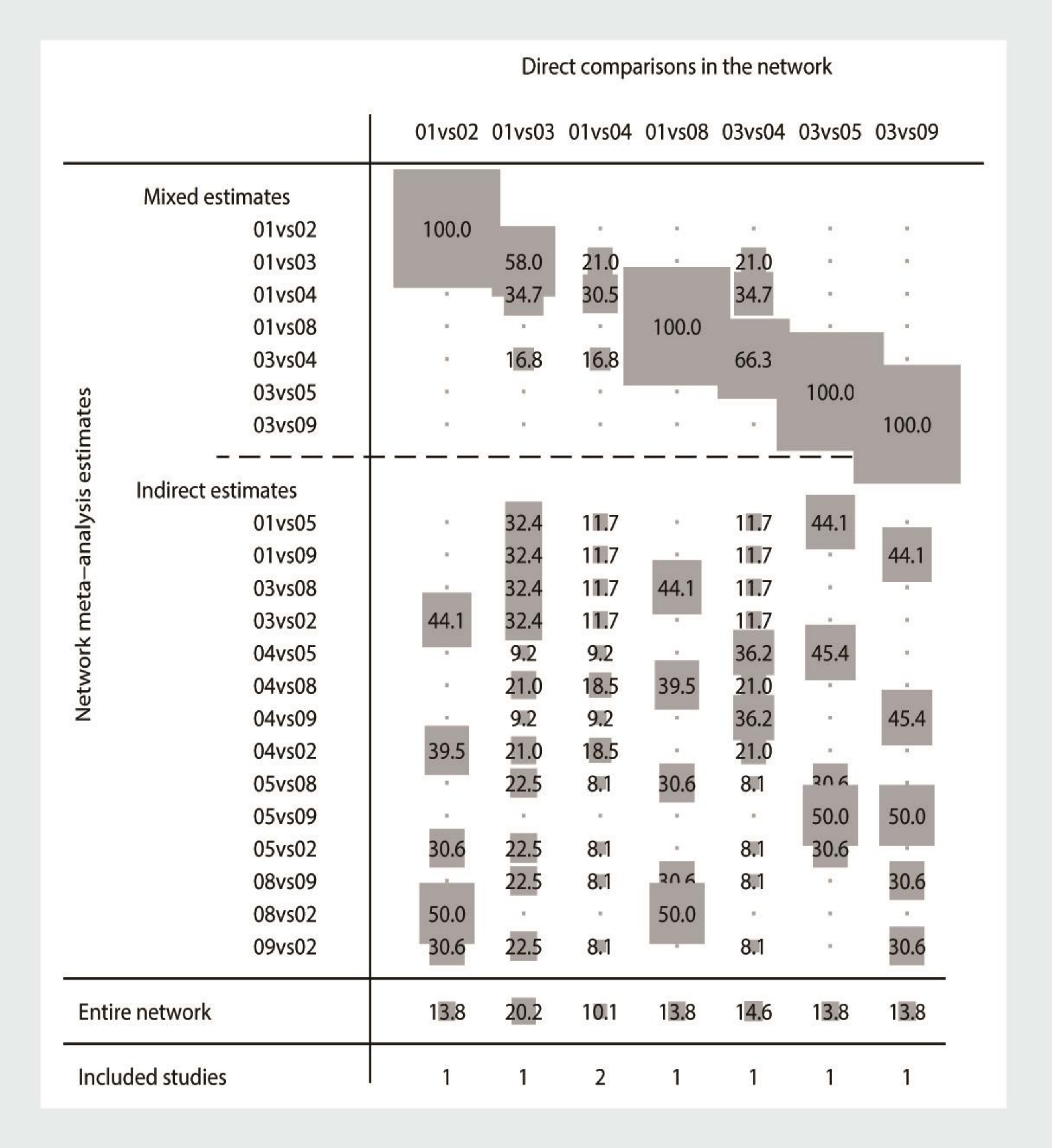

A: FBG

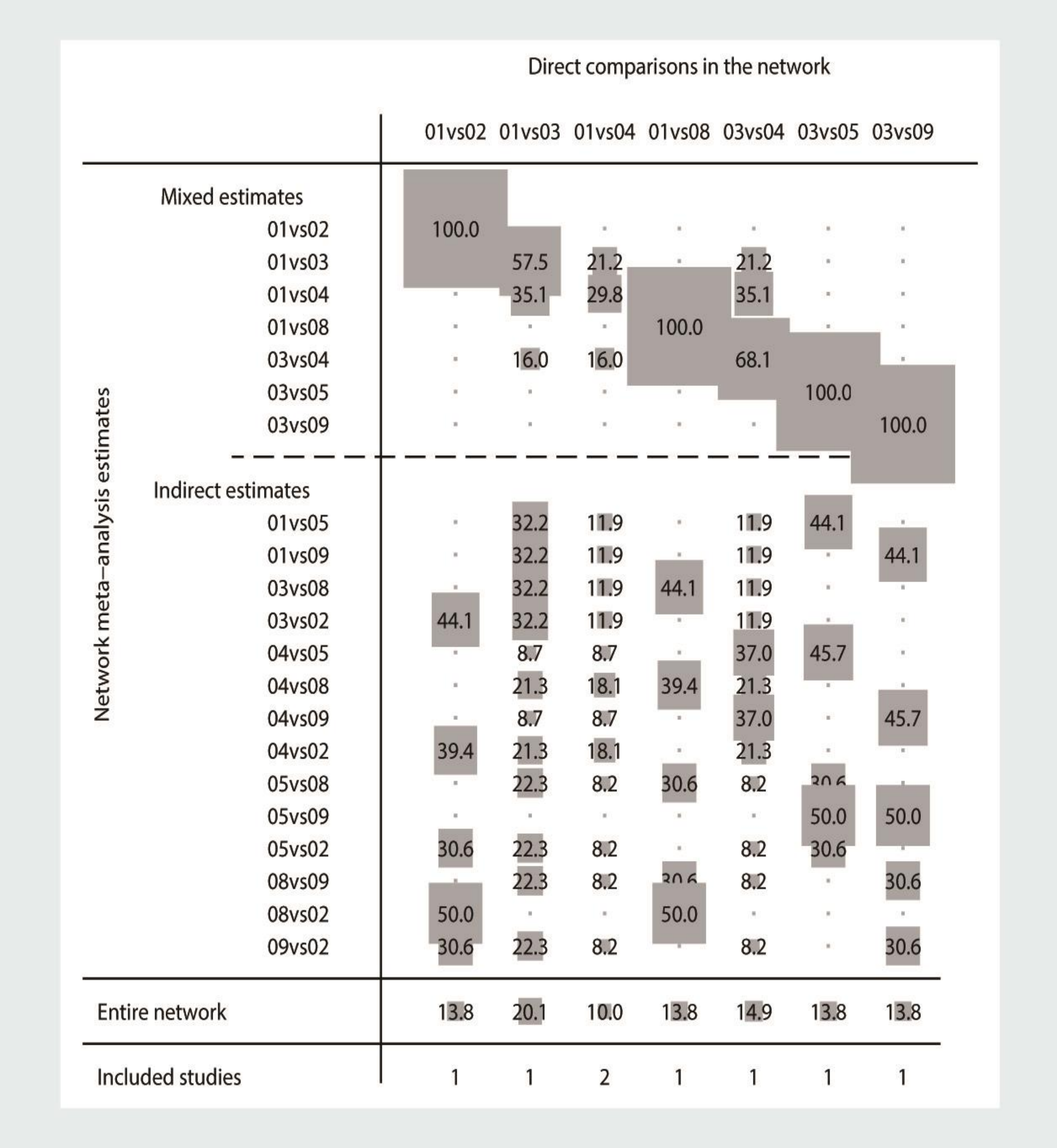

Direct comparisons in the network
01vs02 01vs03 01vs04 01vs08 03vs04 03vs05 03vs09
Network meta-analysis estimates
Mixed estimates
01vs02 100.0
01vs03 57.5 21.2 21.2
01vs04 35.1 29.8 35.1
01vs08 100.0
03vs04 16.0 16.0 68.1
03vs05 100.0
03vs09 100.0
Indirect estimates
01vs05 32.2 11.9 11.9 44.1
01vs09 32.2 11.9 11.9 44.1
03vs08 32.2 11.9 44.1 11.9
03vs02 44.1 32.2 11.9 11.9
04vs05 8.7 8.7 37.0 45.7
04vs08 21.3 18.1 39.4 21.3
04vs09 8.7 8.7 37.0 45.7
04vs02 39.4 21.3 18.1 21.3
05vs08 22.3 8.2 30.6 8.2
05vs09 50.0 50.0
05vs02 30.6 22.3 8.2 8.2 30.6
08vs09 22.3 8.2 8.2 30.6
08vs02 50.0 50.0
09vs02 30.6 22.3 8.2 8.2 30.6
Entire network 13.8 20.1 10.0 13.8 14.9 13.8 13.8
Included studies 1 1 2 1 1 1 1

B: HbA1c

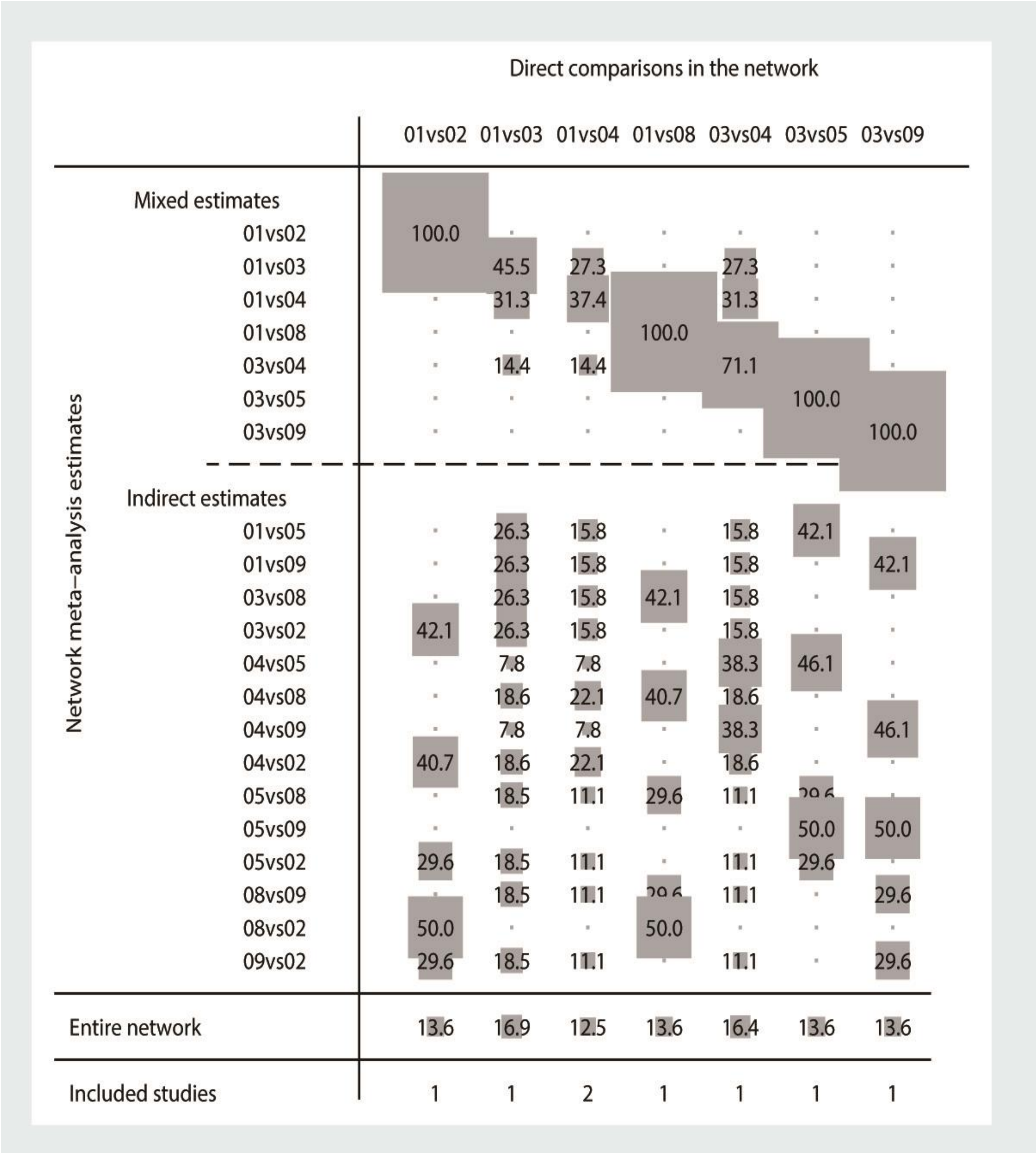
Direct comparisons in the network
01vs02 01vs03 01vs04 01vs08 03vs04 03vs05 03vs09
Network meta–analysis estimates
Mixed estimates
01vs02 100.0
01vs03 45.5 27.3 27.3
01vs04 31.3 37.4 31.3
01vs08 100.0
03vs04 14.4 14.4 71.1
03vs05 100.0
03vs09 100.0
Indirect estimates
01vs05 26.3 15.8 15.8 42.1
01vs09 26.3 15.8 15.8 42.1
03vs08 26.3 15.8 42.1 15.8
03vs02 42.1 26.3 15.8 15.8
04vs05 7.8 7.8 38.3 46.1
04vs08 18.6 22.1 40.7 18.6
04vs09 7.8 7.8 38.3 46.1
04vs02 40.7 18.6 22.1 18.6
05vs08 18.5 11.1 29.6 11.1
05vs09 50.0 50.0
05vs02 29.6 18.5 11.1 11.1 29.6
08vs09 18.5 11.1 11.1 29.6
08vs02 50.0 50.0
09vs02 29.6 18.5 11.1 11.1 29.6
Entire network 13.6 16.9 12.5 13.6 16.4 13.6 13.6
Included studies 1 1 2 1 1 1 1

C: HOMA-β

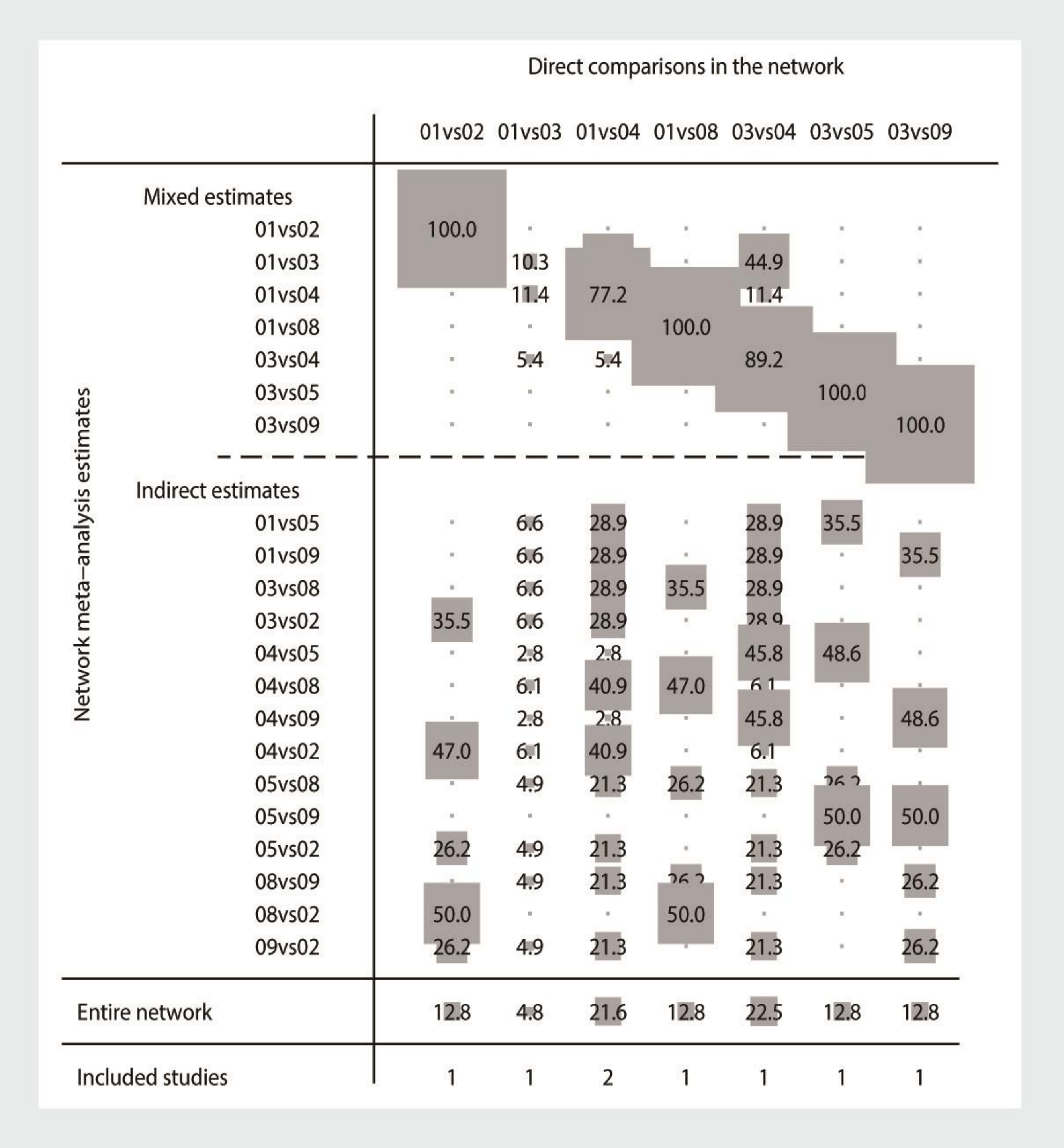

D: Weight loss

1: EXBID; 2: GLI; 3: EXQW; 4: Insulin; 5: MET; 6: PIO; 7: SITA; 8: Placebo; 9: Semaglutide

Figure S6: Node-splitting plots of outcomes

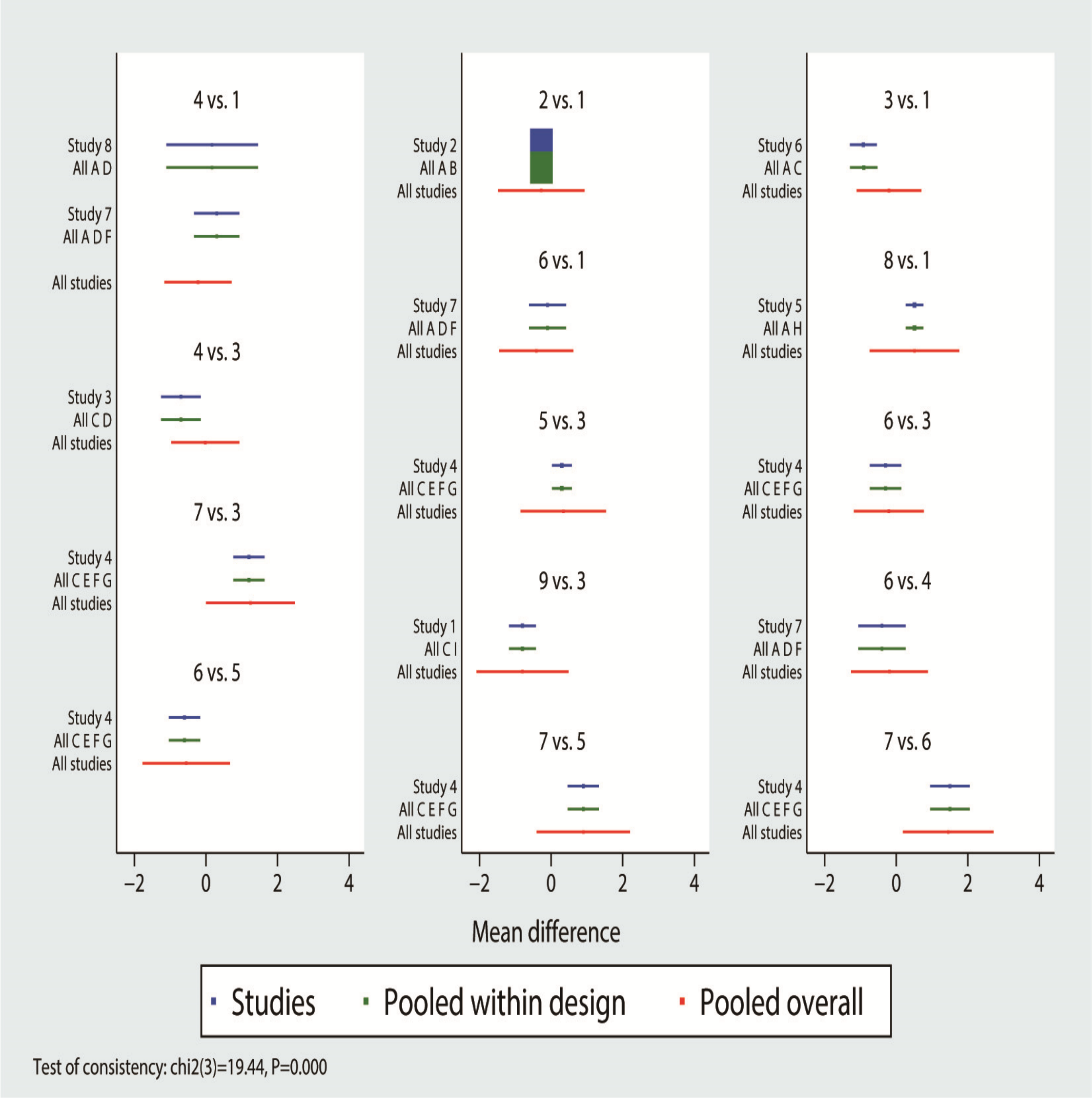

A: FBG

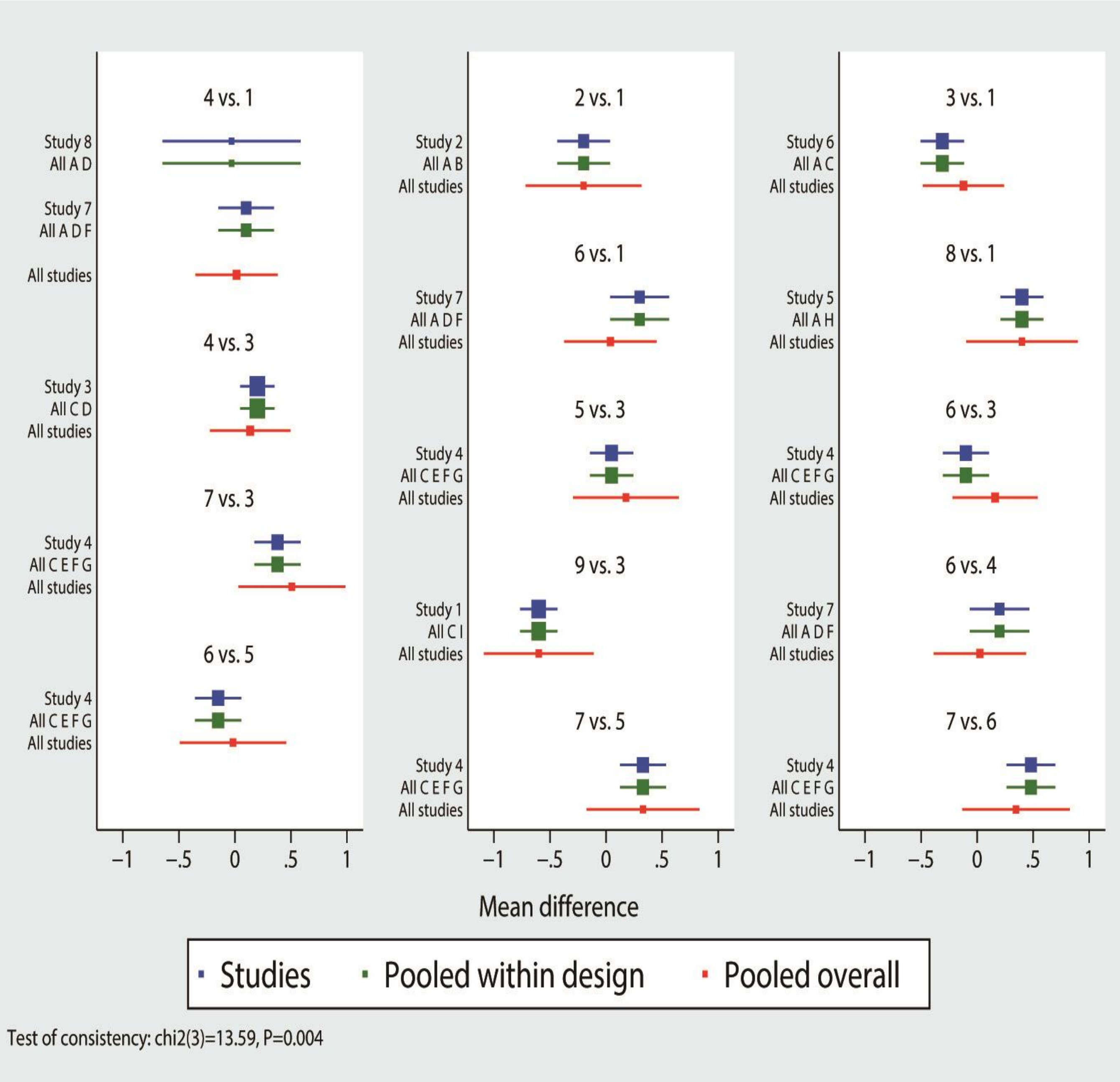

4 vs. 1
Study 8
All A D
Study 7
All A D F
All studies
4 vs. 3
Study 3
All C D
All studies
7 vs. 3
Study 4
All C E F G
All studies
6 vs. 5
Study 4
All C E F G
All studies
2 vs. 1
Study 2
All A B
All studies
6 vs. 1
Study 7
All A D F
All studies
5 vs. 3
Study 4
All C E F G
All studies
9 vs. 3
Study 1
All C I
All studies
7 vs. 5
Study 4
All C E F G
All studies
3 vs. 1
Study 6
All A C
All studies
8 vs. 1
Study 5
All A H
All studies
6 vs. 3
Study 4
All C E F G
All studies
6 vs. 4
Study 7
All A D F
All studies
7 vs. 6
Study 4
All C E F G
All studies
−1
−.5
0
.5
1
Mean difference
Studies
Pooled within design
Pooled overall
Test of consistency: chi2(3)=13.59, P=0.004

B: HbA1c

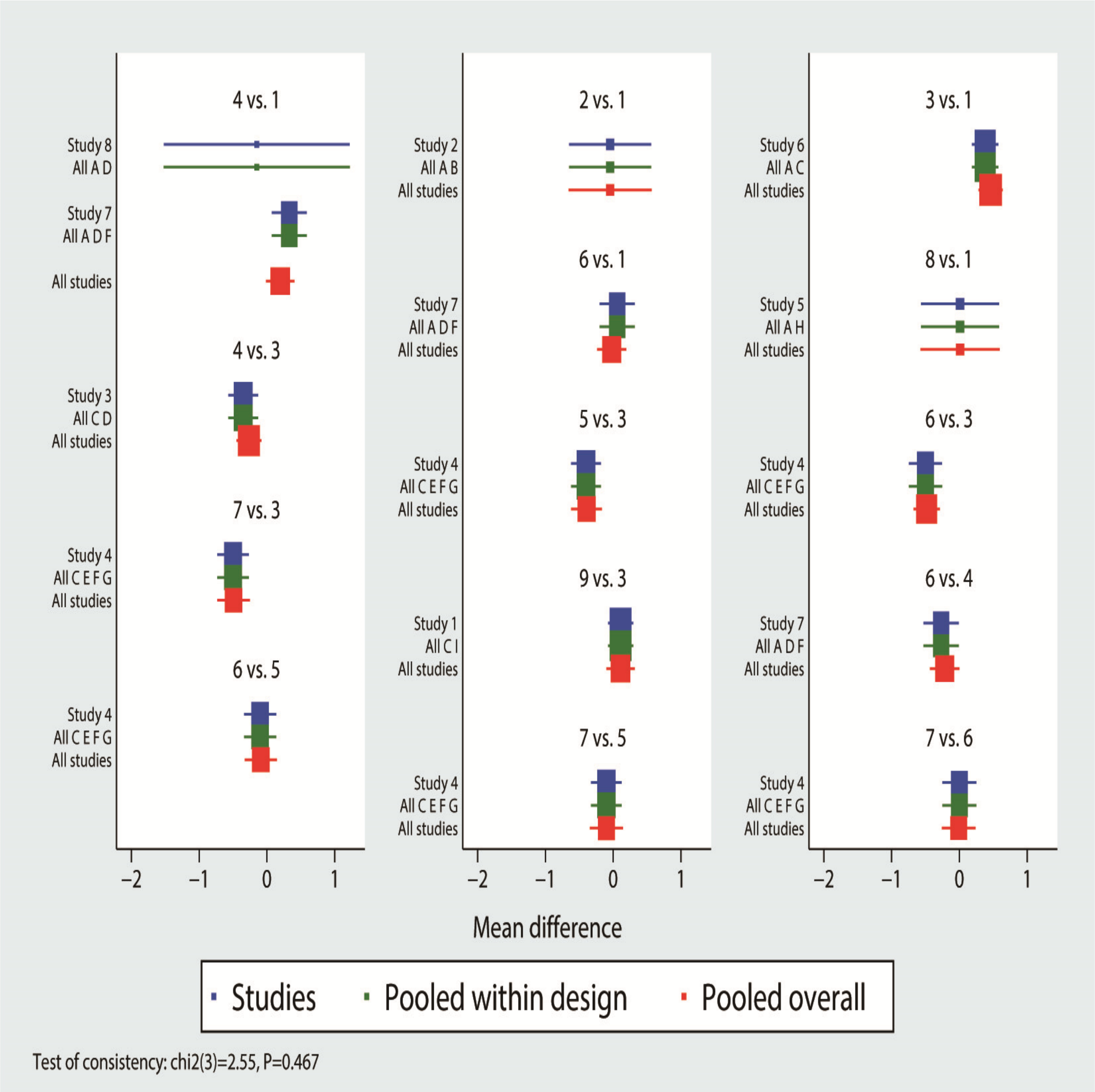
4 vs. 1
Study 8
All A D
Study 7
All A D F
All studies
4 vs. 3
Study 3
All C D
All studies
7 vs. 3
Study 4
All C E F G
All studies
6 vs. 5
Study 4
All C E F G
All studies
2 vs. 1
Study 2
All A B
All studies
6 vs. 1
Study 7
All A D F
All studies
5 vs. 3
Study 4
All C E F G
All studies
9 vs. 3
Study 1
All C I
All studies
7 vs. 5
Study 4
All C E F G
All studies
3 vs. 1
Study 6
All A C
All studies
8 vs. 1
Study 5
All A H
All studies
6 vs. 3
Study 4
All C E F G
All studies
6 vs. 4
Study 7
All A D F
All studies
7 vs. 6
Study 4
All C E F G
All studies
−2
−1
0
1
Mean difference
Studies
Pooled within design
Pooled overall
Test of consistency: chi2(3)=2.55, P=0.467

C: HOMA-β

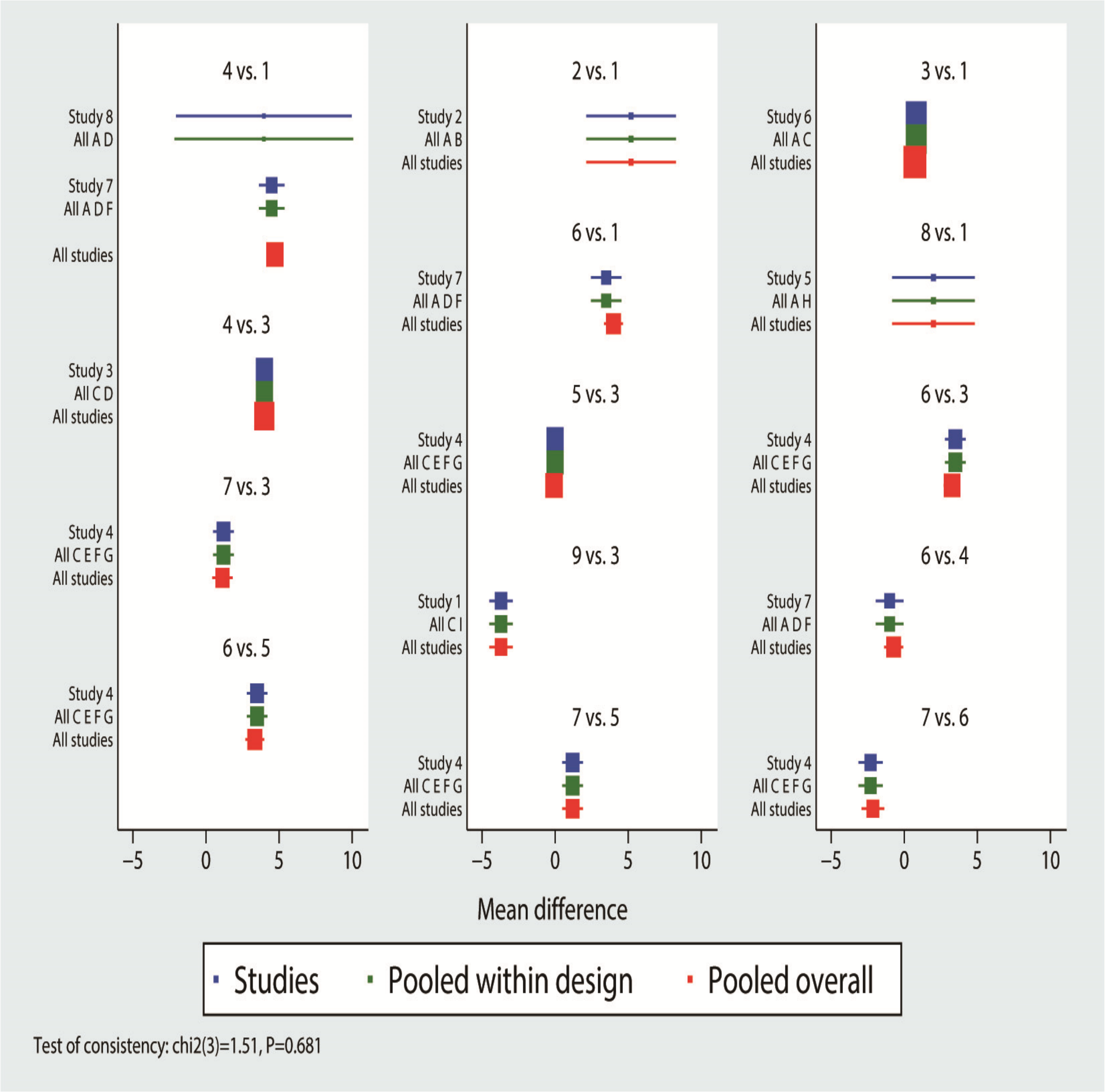

D: Weight loss

1: EXBID; 2: GLI; 3: EXQW; 4: Insulin; 5: MET; 6: PIO; 7: SITA; 8: Placebo; 9: Semaglutide
